# Estrogen rescues muscle regeneration impaired by DUX4 in a humanized xenograft mouse model

**DOI:** 10.1038/s41419-025-07827-2

**Published:** 2025-07-09

**Authors:** Silvia Maiullari, Giada Mele, Patrizia Calandra, Giorgia di Blasio, Sonia Valentini, Alessio Torcinaro, Isabella Manni, Emanuela Teveroni, Fabio Mancino, Luca Proietti, Fabio Maiullari, Maria Pesavento, Ludovica Giorgini, Sabrina Putti, Roberto Rizzi, Sara Bortolani, Ferdinando Scavizzi, Marcello Raspa, Enzo Ricci, Giulia Piaggio, Cesare Gargioli, Alfredo Pontecorvi, Siro Luvisetto, Massimiliano Mazzone, Giancarlo Deidda, Fabiola Moretti

**Affiliations:** 1 Institute of Biochemistry and Cell Biology, National Research Council of Italy, Monterotondo, Italy; 2 PhD program in Sciences of Nutrition, Metabolism, Ageing and Gender Medicine, Catholic University of Rome, Roma, Italy; 3 IRCCS National Cancer Institute Regina Elena, Roma, Italy; 4 Division of Orthopaedic and Traumatology, Fondazione Policlinico Universitario A. Gemelli IRCCS, Roma, Italy; 5 Operative Unit Spinal Surgery, Fondazione Policlinico Universitario A. Gemelli IRCCS, UCSC, Roma, Italy; 6 National Institute of Molecular Genetics, Milano, Italy; 7 Institute of Physical Chemistry – Polish Academy of Sciences, Warsaw, Poland; 8 Department of Medical-Surgical Science and Biotechnologies, “Sapienza” University of Rome, Latina, Italy; 9 Complex Operational Unit of Neurology, Fondazione Policlinico Universitario A. Gemelli IRCCS, Roma, Italy; 10 Department of Neuroscience, Catholic University of Rome, Roma, Italy; 11 Department of Biology, Rome University Tor Vergata, Roma, Italy; 12 Department of Medicine and Translational Surgery, Catholic University of Roma, Roma, Italy; 13 Laboratory of Tumor Inflammation and Angiogenesis, Center for Cancer Biology, VIB, Leuven, Belgium; 14 Laboratory of Tumor Inflammation and Angiogenesis, Center for Cancer Biology, Department of Oncology, KU Leuven, Leuven, Belgium

**Keywords:** Neuromuscular disease, Translational research, Muscle stem cells, Mesenchymal stem cells

## Abstract

Facioscapulohumeral dystrophy (FSHD) is an autosomal dominant muscular dystrophy and one of the most frequent hereditary myopathies. The pathology shows a wide range of clinical signs, with modifying factors contributing to this variability, especially in patients with mild disease. Among these factors, the beneficial activity of estrogen hormones is controversial. We investigated the effect of 17β-estradiol (E_2_) and the 5α-dihydrotestosterone-derived 3β-androstenediol (3β-diol) on muscle regeneration. To recapitulate human hormone sensitivity, we developed a humanized heterokaryon FSHD mouse model by engrafting human immortalized myoblasts or human primary muscle mesenchymal stromal cells into surgically treated murine muscle. Inducible lentiviral expression of the pathogenic FSHD gene, DUX4, in human cells impaired the structural and functional recovery of murine muscle, providing a humanized mouse model of DUX4-mediated pathogenicity and proving that the biological effect of DUX4 spreads across the neighbouring murine nuclei. Both hormones counteracted DUX4 transcriptional activity and rescued structural and functional muscle performance impaired by DUX4 expression, while being inefficient in control grafts. The beneficial activity of estrogen in this heterokaryon model supports the hypothesis that these hormones contribute as a modifying factor in FSHD.

## Introduction

Facioscapulohumeral dystrophy (FSHD) is a complex and heterogeneous disease common in adulthood with a prevalence of 5–12 in 100,000 individuals [1]. It is characterized by progressive muscle weakness and wasting, which can eventually lead to a wheelchair situation [2]. FSHD is caused by epigenetic derepression of the D4Z4 microsatellite repeat array at the 4q35 region, which activates the primate-specific double homeobox protein 4 gene (DUX4) transcription [3–5]. DUX4 represents the primary pathogenic factor of the disease, affecting numerous cellular processes, including muscle differentiation and cell death [6].

In 95% of FSHD patients, derepression is caused by a partial deletion of the array to a critical number of D4Z4 units [1–10] (FSHD1, OMIM:158900). This genetic defect contributes to FSHD variability: residual repeats are roughly inversely correlated with disease severity [7, 8]. However, mutation carriers within the 7–10 D4Z4 units range show the highest intra- and interfamilial clinical variability, suggesting that additional modifying factors impact clinical manifestation [3, 6, 9]. Various epidemiological studies have reported a significantly higher proportion of affected males than females, especially in medium- to long-allele carriers, with increased asymptomatic carriers or less severe symptoms or later onset in females compared to males [10–17]. A reduced correlation between fragment size and age-corrected clinical severity score in female patients supports the existence of disease modifiers in women [17]. A large-scale genotype-phenotype analysis reported a different age-specific cumulative risk of muscle impairment dependent on sex, with women showing a decreased risk in the age ranges 15–20 and 50–60 years, suggesting the hypothesis of a possible role of estrogen [15]. Accordingly, some clinical studies reported symptoms worsening in women undergoing hormone drop following hysterectomy/ovariectomy or hormone therapy in cancer [8, 18]. Not all population studies reported gender differences, although in some cases, the lack of age classes could mask the estrogen status [8, 19]. Eventually, studies on prevalence and disease progression did not evidence a contribution of estrogen exposure time to the disease [20]. Thus, the role of estrogen as a potential FSHD disease modifier remains controversial.

In vitro, estrogen antagonizes DUX4 activity in myoblasts derived from FSHD patients [18, 21]. In vivo, FSHD animal models did not directly address the consequences of hormone treatment. Most FSHD models are based on the exogenous expression of the human DUX4 in murine muscle and recapitulate DUX4 toxicity [22–27]. However, the high levels of DUX4 in these models may hide the impact of modifier factors. In fact, in FSHD muscle, DUX4 mRNA is expressed in 0.8 ÷ 1 out of 1000 nuclei [28], and the protein in 1 out of 1000 ÷ 2000 nuclei [29], thus associating the disease with sporadic expression of DUX4. Interestingly, the inducible FLExDUX4 mouse, which in the uninduced state expresses low leaky DUX4 levels, develops a delayed phenotype in female mice [30], disappearing by 12 months, arguing for the possible involvement of reproductive senescence in mice. In the same model, DUX4 induction by tamoxifen, an estrogen receptor antagonist, causes a more severe phenotype in FLExDUX4 females compared to their male littermates [25].

We investigated the effect of estrogen on DUX4 by treating animals with two estrogens: 17β-estradiol (E_2_) and 3β-androstenediol (3β-diol). Estrogen activity is mainly driven by two nuclear receptors, the estrogen receptors α and β (ERα and ERβ). E_2_ is the predominant estrogen in premenopausal females and binds to ERα and ERβ with similar affinity [31]. Three β-diol is produced by the hydroxylation of dihydrotestosterone (DHT), possesses a higher affinity for ERβ than ERα [31], and its blood levels are up to 4.5-fold higher in males compared to females, also depending on the age [32]. Since in FSHD myoblasts, DUX4 transcriptional activity is reduced mainly by ERβ [18], using 3β-diol in vivo may indicate ERβ involvement.

To mimic FSHD and recapitulate the low number of DUX4-expressing cells in the muscle, we developed an orthotopic xenograft of human muscle-derived cells in the murine Tibialis Anterior (TA) muscle. This model provides more accurate information about hormone activity in human cells, as mice and humans express different ER variants [33].

We transplanted immortalized myoblasts (ImMyobs) and primary muscle mesenchymal stromal cells (MMSCs). MMSCs act as a “supporting group” in muscle regeneration [34]. MMSCs, especially perivascular cells, synergize in vivo with muscle stem cells (MuSCs, also known as satellite cells), forming more myofibers than those obtained by sole MuSCs transplantation [35] and differentiating into muscle cells [36, 37]. Accordingly, their transplantation in mice has been employed to study muscle regeneration [36, 38–40]. In FSHD, MMSCs contribute to FSHD muscle degeneration [41], and in FSHD muscle biopsies, perivascular inflammation and a reduction in capillary density have been observed [42, 43], suggesting an impairment of the muscle stroma.

We prove that DUX4 expression impairs murine muscle regeneration, spreading its activity to murine nuclei. Both E_2_ and 3β-diol antagonize DUX4 transcriptional activity, counteract its toxicity, reduce DUX4-induced muscle fibrosis, and improve muscle regeneration, providing valuable insights into the function of these hormones in human FSHD pathology.

## Results

### DUX4 is active in human MMSCs

To study the effect of estrogen on human DUX4, we exploited an orthotopic xenograft of human cells. We transplanted immortalized myoblasts (ImMyobs) or primary muscle mesenchymal stromal cells (MMSCs), engineered to express inducible DUX4.

**Fig. 1:**
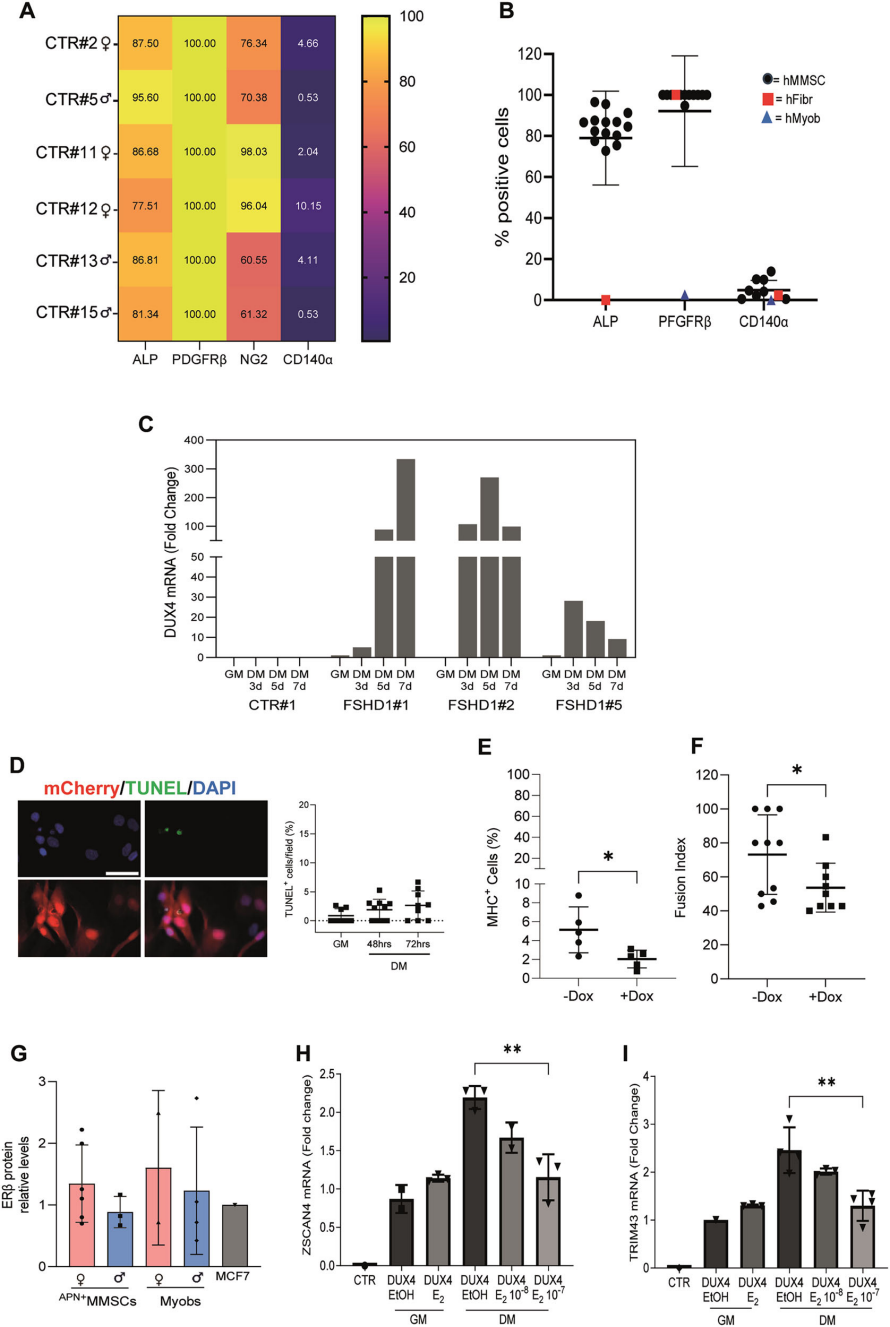
DUX4 is active in human MMSCs. Heat map (**A**) and cumulative graph (**B**) of alkaline phosphatase (ALP), platelet-derived growth factor receptor beta (PDGFRβ), neural/glial antigen 2 (NG2) (only in **A**), and platelet-derived growth factor receptor alpha (PDGFRα or CD140α) positive human MMSCs isolated from different healthy subject biopsies (CTR). Human fibroblasts (hFibr) and myoblasts (hMyob) served as controls. **C** DUX4 mRNA levels by RT-qPCR in ^APN+^MMSCs from biopsies of 1 CTR and 3 FSHD1 patients. ^APN+^MMSCs were collected in growth (GM) or differentiating medium (DM) at the indicated time points. All samples were normalized to hGAPDH and relative to the DUX4 value in GM FSHD1#1, set to 1. **D** Representative image of TUNEL^+^ DUX4-^APN+^MMSCs infected with mCherry and doxycycline-inducible DUX4 lentiviral vectors upon Dox treatment under growing conditions. The graph on the right reports the percentage of TUNEL^+^ cells/field in GM or the differentiation medium (DM) at the indicated time points. At least 9 fields from 2 biological replicates were counted. Scale bar is 5 µm. **E** Percentage of MHC^+^ cells/field in DUX4-^APN+^MMSCs grown in DM for 5 days and treated ±Dox (1 µg/ml) (nuclei in MHC^+^ cells/total number of nuclei). Five fields from 2 biological replicates were counted. Data is shown as mean ± SD, *P < 0.05, unpaired *t*-test. **F** Fusion index in DUX4-^APN+^MMSCs grown in DM for 7 days and treated ±Dox (percentage of nuclei in MHC^+^myotube, where a myotube is a MHC^+^ cell with at least 3 nuclei). Ten fields from two biological replicates were counted. Data is shown as mean ± SD, *P < 0.05, unpaired *t*-test. **G** Quantification of ERβ protein levels in CTR-^APN+^MMSCs and human myoblasts from healthy subjects. MCF7 cells were used as a positive control, and their value was arbitrarily set to 1. The relative blots are shown in Fig. S1G. TRIM43 (**H**) and ZSCAN4 (**I**) mRNA levels by RT-qPCR in ^APN+^MMSCs overexpressing DUX4 and collected in GM or after 48 h in DM and treated as indicated. CTR is non-transfected ^APN+^MMSCs. All samples were normalized to hGAPDH. *n* = 3 replicates/condition. Data is shown as mean ± SD., **P < 0.01, one-way ANOVA with Dunnett’s multiple comparisons test.

Since no single molecular marker can exclusively identify MMSC subpopulations, and there is no consensus on unambiguous criteria for their identification [44], we isolated human MMSCs (hMMSCs) from muscle biopsies by selective enzymatic dissociation and culture conditions, and characterized them using human fibroblasts and human myoblasts as control [45, 46]. Heat map of a representative group of cells isolated from healthy individuals (CTR) (Fig. 1A and Table S1) and cumulative analysis (Fig. 1B) show high positivity of these cells to alkaline phosphatase activity (ALP) (Fig. S1A) and platelet-derived growth factor receptor beta (PDGFRβ) (Fig. S1B), independently of the sex (Fig. 1A), resembling molecular features of geographically located perivascular cells [45, 47]. The almost absence of PDGFRβ in human myoblasts (less than 2% positive cells) (Figs. 1B and S1B) and the nearly 100% positivity in hMMSCs (Figs. 1A, B and S1B) suggest the absence of human muscle stem cells (MuSCs) in the populations, in agreement with the mild enzymatic dissociation of the tissue. The positivity to platelet-derived growth factor receptor alpha (PDGFRα, also CD140α) suggests the presence of fibroadipogenic progenitors (FAPs) (Fig. 1A, B and Table 1). However, double staining with CD140α and NG2, a marker absent in FAPs [48], indicates the co-expression of both markers, excluding that they are canonical FAPS, possibly belonging to an endothelial-like population (Fig. S1C and Table 1). Since the isolated MMSC populations are composed of ALP^+^/PDGFRβ^+^/NG2^+^/PDGFRα^−^cells with an estimated abundance of ≥80% (Fig. 1B), they will be referred to as ^APN+^MMSCs. These cells express desmin (Fig. S1D) and form myotubes in vitro (Fig. S1E) [37], supporting their contribution to myofiber regeneration.

**Table 1:** NG2 and CD140α double staining in the indicated cell types and selected ^APN+^MMSCs from biopsies of healthy subjects (CTR).

CELL TYPE	CD140α^−^/NG2^+^	NG2^−^/CD140α^+^	NG2^+^/CD140α^+^
CD56^+^hMyoblast	1.61	0.04	0
hFibroblast	20	1.84	0.56
CTR#2 ♀	73	1.32	3.34
CTR#5 ♂	70	0.01	0.38
CTR#11 ♀	95.66	0.04	1.99
CTR#12 ♀	86.30	0.41	9.74
CTR#13 ♂	60.55	1.56	2.55
CTR#15 ♂	60.91	0.12	0.41

The numbers report the percentage of indicated cells.

Interestingly, ^APN+^MMSCs isolated from the muscle biopsies of three FSHD1 patients (Table S1) express DUX4 mRNA when primed for muscle differentiation (Fig. 1C), with a pattern of expression analogous to that observed in myoblasts of FSHD patients [18, 49]. Conversely, DUX4 mRNA levels were not detectable in CTR ^APN+^MMSCs (CTR#1) (Fig. 1C). These data support the function of active myogenic enhancers on the DUX4 promoter during muscle differentiation [50].

To prove whether ^APN+^MMSCs sustain DUX4 activity, ^APN+^MMSCs were infected with lentiviral vectors carrying doxycycline-inducible HA-DUX4 (DUX4-^APN+^MMSC) and the mCherry fluorescent marker. Since high levels of DUX4 cause considerable cell death [51–53], we used a low multiplicity of infection to limit DUX4 expression. Indeed, upon doxycycline (Dox) treatment, a low number of TUNEL^+ APN+^MMSCs was observed under growth conditions (GM), with a slight increase during muscle differentiation (DM) when the nuclear localization and activity of DUX4 increased [18, 23], without exceeding 10% (Fig. 1D), sustaining the low toxicity of the expressed DUX4. DUX4 also impairs muscle differentiation of FSHD myotubes. Testing the differentiation marker myosin heavy chain (MHC), we observed reduced muscle differentiation of DUX4-expressing ^APN+^MMSCs (Fig. 1E, F) upon Dox treatment. Thus, DUX4 shows pathogenic features in ^APN+^MMSCs that resemble those observed in myoblasts. The levels of LEUTX, a DUX4 target, were lower in the uninduced state (i.e., Dox) than in myotubes from FSHD patients and were highly induced upon Dox treatment (Fig. S1F), which is associated with DUX4 induction (Fig. S1G), demonstrating the low leakiness of the system and confirming the transcriptional activity of DUX4.

We then tested whether DUX4 activity is controlled by estrogen in ^APN+^MMSCs, as in myotubes [18]. The estrogen receptor beta (ERβ) protein levels are similar between ImMyobs and ^APN+^MMSCs cells, regardless of sex, and are comparable to those in the ERβ^+^ human breast cancer cell line, MCF7 (Figs. 1G and S1H). Conversely, ERα is almost undetectable at protein levels, and its mRNA is very low compared to MCF7 independently of sex (Fig. S1H, I). Following DUX4 expression, the mRNA levels of DUX4 targets TRIM43, ZSCAN4 and MBD3L2 were reduced by treatment with 17β-estradiol (E_2_) during muscle differentiation (DM) in a dose-response manner (Figs. 1H, I and S1J), whereas they were not altered in the proliferation phase (GM) as observed in myoblasts [18]. Therefore, estrogen antagonizes DUX4 activity in ^APN+^MMSCs, supporting their use to model FSHD conditions.

### DUX4 impairs the contribution of human cells to murine muscle regeneration

To set up the human cell xenografts, we compared the transplantation efficiency of primary ^APN+^MMSCs and ImMyobs. A few myofibers were removed from the TA muscle of NOD scid gamma male mice (NSG), and 1 × 10^6^ human cells were layered in the created pocket (Fig. S2A–C). This model mimics partial volumetric muscle loss (VML) and allows the evaluation of the contribution of human cells to murine muscle regeneration [54, 55]. Human ^APN+^MMSCs or ImMyobs were enclosed within Matrigel, free of estrogen-like substances, to increase cell survival and ensure homogeneous cell layering. For each graft, a mix of ^APN+^MMSCs or ImMyobs was used to limit the interference of individual features. Moreover, since no difference in the levels of ERβ and ERα (see Fig. 1G) or in hormone sensitivity was previously observed between female and male myoblasts [18], the cell mix was always composed of cells from both sexes and implanted in male NSG unless otherwise specified.

**Fig. 2:**
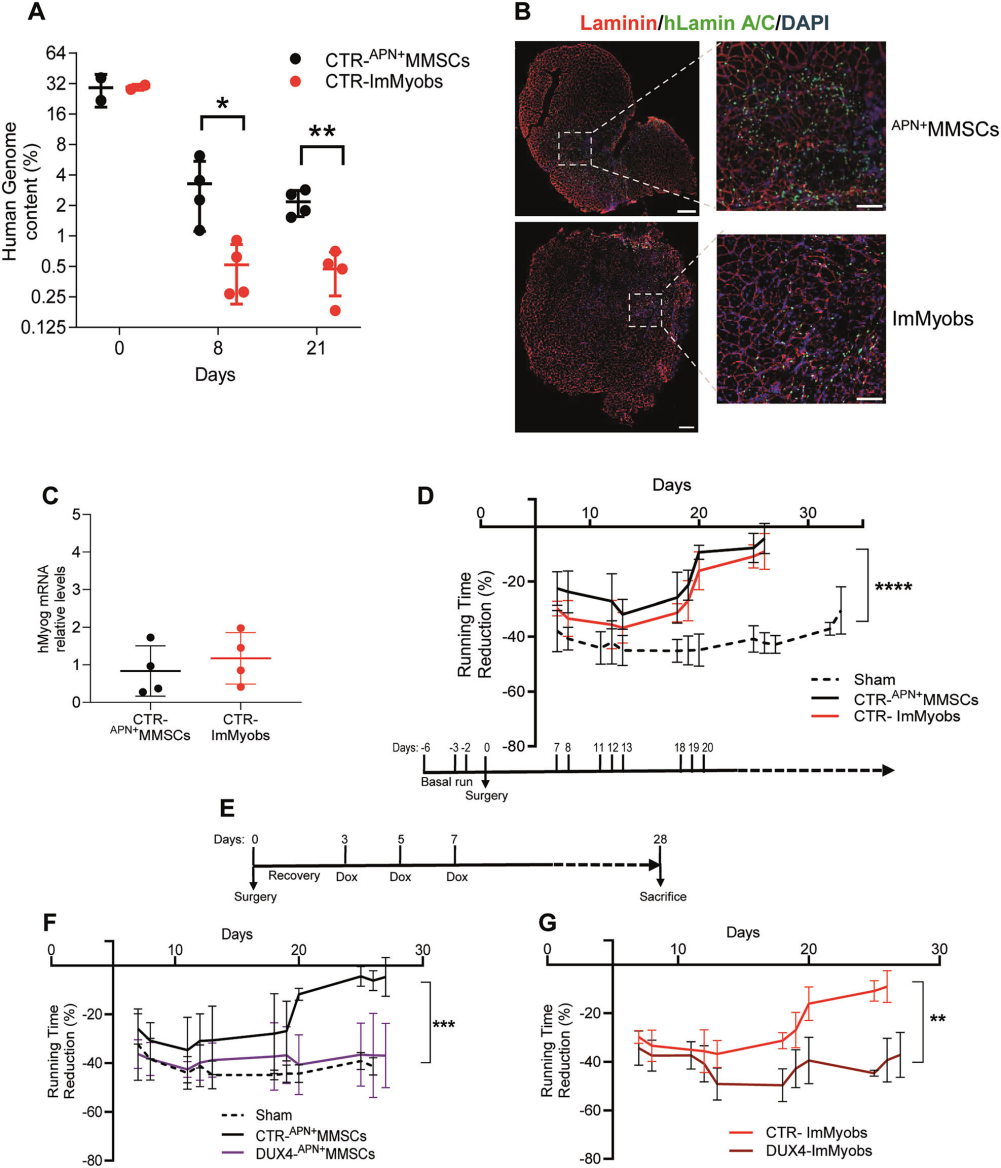
DUX4 impairs the contribution of human cells to murine muscle regeneration. **A** Percentage of human genome content in male CTR-^APN+^MMSCs or CTR-ImMyobs engrafts and collected at the indicated time points. Human genome was calculated relative to an absolute standard curve, using FOXP1 gene primer pairs. Data is from one experiment, *n* = 4 mice/group. Data is shown as mean ± SD. *P < 0.05, **P < 0.01, multiple unpaired *t*-test. **B** Representative images of TA muscle grafts obtained by transplantation of 1 × 10^6^ CTR-^APN+^MMSCs or CTR-ImMyobs. Mice were sacrificed 4 weeks following transplantation. Laminin stains myofibers (red), human Lamin A/C stains human nuclei (green), and DAPI counterstains nuclei (blue). On the left, higher magnification panels. Scale bar of the right panels is 250 µm. **C** Human Myogenin (hMyog) mRNA levels by RT-qPCR in CTR-^APN+^MMSCs or CTR-ImMyobs grafts and collected 28 days after surgery. All samples were normalized to hGAPDH and relative to the mean value of a group of CTR-ImMyobs arbitrarily set to 1. Data is from 1 experiment, *n* = 4 mice/group. Data is shown as mean ± SD. **D** Summary of treadmill test data performed with male mice undergoing surgery and transplanted with sole matrigel (Sham) or CTR-^APN+^MMSCs or CTR-ImMyobs, according to the scheme reported below the graph. The reduction was calculated relative to the mean of a 3-day basal-run test for each mouse. Data is from three independent experiments for Sham and one for CTR- ^APN+^MMSCs and -ImMyobs. *n*_sham_ = 10, n_APN+MMSCs_ = 4, *n*_ImMyobs_ = 4. Data is shown as mean ± SD, ****P < 0.0001, mixed effects model ANOVA (cell type factor *p* < 0.0001, matching *p* < 0.0001, no equal variability assumption). **E** Scheme of animal treatment. Summary of treadmill test data performed with male animals undergoing surgery and transplanted with sole matrigel (Sham) or transplanted with CTR-^APN+^MMSCs or DUX4-^APN+^MMSCs (**F**) or CTR-ImMyobs or DUX4-ImMyobs (**G**). The reduction was calculated relative to the mean of a 3-day basal-run test for each mouse. Data is from three independent experiments for MMSCs *n*_sham_ = 8, *n*_CTR-MMSCs_ = 8, *n*_DUX4-MMSCs_ = 6, and one experiment for ImMyobs. *n*_CTR-ImMyobs_ = 4, *n*_DUX4-ImMyobs_ = 4. Data is shown as mean ± SD. **P < 0.01, ***P < 0.001, mixed effects model ANOVA (F, cell type factor *p* = 0.0004, matching *p* < 0.0001; G, cell type factor *p* = 0.009, matching *p* < 0.0001, no equal variability assumption).

DNA analysis revealed that 1 × 10^6^ human cells represent ~30% of the entire murine TA muscle genomes. Eight days after transplantation, both ^APN+^MMSCs and ImMyobs from healthy individuals (CTR-^APN+^MMSCs and CTR-ImMyobs) underwent a rapid drop (Fig. 2A), with a 6-fold lower survival of ImMyobs compared to ^APN+^MMSCs (Fig. 2A). Both human cell types integrated into murine myofibers, forming heterokaryon mixed myofibers (Fig. 2B), and single cells expressed similar levels of human myogenin (Fig. 2C). The treadmill assay showed that CTR- ^APN+^MMSCs and -ImMyobs transplanted mice regained their running rate almost entirely within 25 days (Fig. 2D). In contrast, surgery-treated mice implanted with sole Matrigel (sham group) started to recover after 30 days (Fig. 2D). CTR-ImMyobs transplanted mice recovered more slowly than those transplanted with ^APN+^MMSCs, in agreement with their reduced number (Fig. 2D, red line). Transplantation of human fibroblasts was ineffective in recovery and even worsened it due to massive muscle fibrosis (Fig. S2D, E), confirming that only human muscle-committed cells contribute to murine muscle regeneration.

We then tested the effect of Dox-inducible DUX4 expression in this system. To avoid the interference of DUX4 activity during cell engraftment, doxycycline was given subcutaneously (sc) to the mice 3 days after surgery and subsequently every other day for 28 days (Fig. 2E). Engraftment of DUX4-^APN+^MMSCs resulted in the formation of human-murine heterokaryons as well (Fig. S2F). However, DUX4-^APN+^MMSCs and DUX4-ImMyobs were unable to sustain the functional recovery of transplanted mice in the same time frame as CTR-^APN+^MMSCs and CTR-ImMyobs (Fig. 2F, G). Thus, DUX4 delays the murine muscle regeneration in this heterokaryon model.

### Estrogen antagonizes DUX4 transcriptional activity

**Fig. 3:**
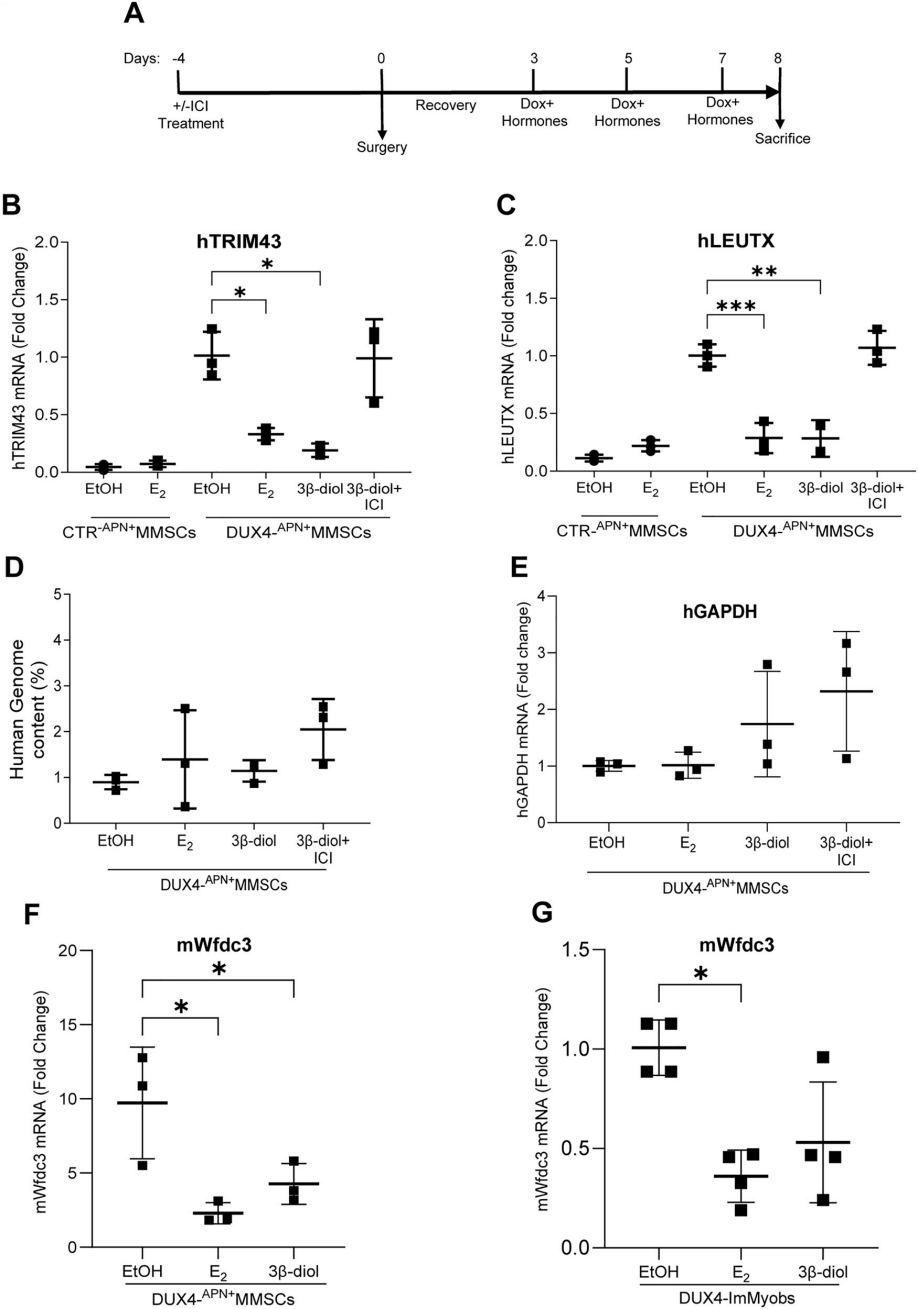
Estrogen antagonizes DUX4 transcriptional activity. **A** Scheme of short-term animal treatment. Vehicle (EtOH), E_2_ (100 µg/kg body weight), 3β-diol (1 mg/kg bw), ICI (200 mg/kg bd). Human TRIM43 (**B**) and LEUTX (**C**) mRNA levels by RT-qPCR in male CTR- or DUX4- ^APN+^MMSCs grafts treated as indicated. All samples were normalized to hAct and relative to EtOH-treated DUX4 transplanted animals, whose mean was arbitrarily set to 1. *n* = 3 mice/group. Data is shown as mean ± SD. Data is from 1 experiment, *P < 0.05, **P < 0.01, ***P < 0.001. One-way ANOVA with Dunnett’s multiple comparisons test. **D** Human DNA quantification by q-PCR in the TRIzol DNA fractions of the same samples as in (**B**, **C**). Human genome was calculated relative to an absolute standard curve, using FOXP1 gene primer pairs. *n* = 3 mice/group. **E** Human GAPDH mRNA levels by RT-qPCR in TA muscles of the same samples as in (**A**, **B**). Samples were normalized to murine GAPDH. Murine Wfdc3 mRNA levels by RT-qPCR in TA muscle of male DUX4- ^APN+^MMSCs (**F**) or DUX4-ImMyobs grafts (**G**), treated as indicated. All samples were normalized to murine GAPDH. The Wfdc3 levels were relative to those of the contralateral leg of each mouse (**F**) or to EtOH-treated mice arbitrarily set to 1 (**G**). Data are from muscles as in (**B**, **C**), *n* = 3 mice/group (**F**). Data is from one experiment, *n* = 4 mice/group (**G**). Data is shown as mean ± SD. *P < 0.05, One-way ANOVA with Dunnett’s multiple comparisons test.

Based on previous results, we investigated whether estrogen could correct the pathogenicity of DUX4. DUX4 transcriptional activity is crucial for its pathogenicity, and estrogen in vitro antagonizes this activity [18, 21]. Given the enhanced activity of DUX4 in the early phases of muscle differentiation [18, 56, 57], we measured human DUX4 targets in the xenografted TA samples 8 days after cell transplantation, when myofiber differentiation was not completed [58]. NSG mice were treated with E_2_, 3β-diol, or vehicle (EtOH) plus Dox according to the indicated scheme (Fig. 3A), using hormone doses that resemble human levels [59]. To confirm the role of ER, a group of animals was pretreated with the selective estrogen receptor degrader (SERD), ICI (ICI182780, fulvestrant), which induces the downregulation of ERβ also in human muscle cells (Figs. 3A and S3A) [60].

DUX4 expression significantly increased the mRNA of its human targets compared to CTR-^APN+^MMSCs engrafted muscle (Figs. 3B, C and S3B–D). E_2_ and 3β-diol significantly reduced these levels in DUX4 engrafted muscles, whereas E_2_ was ineffective in CTR mice (Figs. 3B, C and S3B, C). Co-treatment of animals with 3β-diol and ICI rescued these target levels, confirming ERβ as the primary mediator of hormone inhibitory activity. In the same samples, human DNA levels and GAPDH mRNA levels were not substantially altered by hormone treatments (Fig. 3D, E), indicating that the hormones had no significant effect on cell number or survival but rather interfered specifically with DUX4 activity.

DUX4 pathogenicity is also attributed to its ability to spread from one nucleus into nearby ones of the same myofiber [61]. Given the presence of mixed human-murine myofibers, we tested whether human DUX4 could induce its murine targets. The levels of Wfdc3, a murine target of DUX4 [22], were increased in DUX4 engraftments compared to the healthy contralateral leg (Fig. 3F), and E_2_ and 3β-diol significantly reduced these levels (Fig. 3F). Similar findings were observed in ImMyobs-transplanted mice (Fig. 3G). These data indicate that DUX4 diffuses from human into the murine nuclei of the myofibers in which human cells are integrated, expanding its pathogenicity to the murine muscle and providing a plausible explanation for the functional impairment of mice engrafted with DUX4-expressing human cells.

### Estrogen antagonizes DUX4-mediated toxicity

**Fig. 4:**
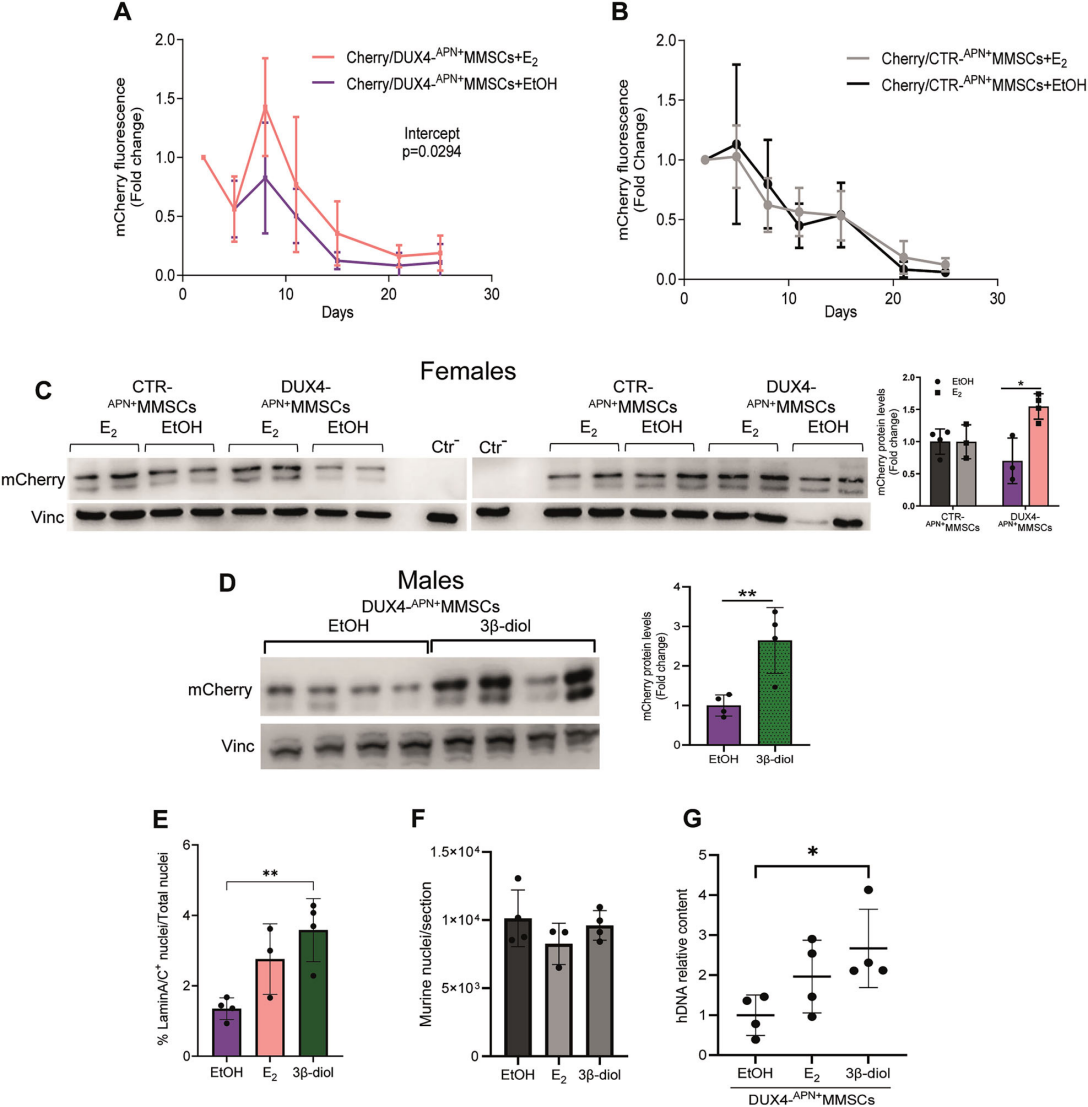
Estrogen antagonizes DUX4-mediated toxicity. Quantification of mCherry fluorescence signal by TA muscle of female mice transplanted with DUX4-^APN+^MMSCs (**A**) or CTR-^APN+^MMSCs (**B**) and treated with E_2_ or EtOH according to the scheme in Fig. 2E. Photon emission was measured as photons per second (*p*/*s*). Data is from one experiment, *n* = 6 mice/group. Data is shown as mean ± SD. *P < 0.05, Linear regression: Intercept values, *F* = 4891 DFn = 1 DFd = 88. WB analysis of mCherry protein in DUX4- or CTR-^APN+^MMSCs grafts of female (**C**) animals as in **A**, **B**, or male animals (**D**) treated as indicated. Animals were sacrificed five (**C**) or 4 weeks (**D**) after transplantation. The graphs on the right report the relative quantification of WB. All samples were normalized to murine Vinculin. **D** *n* = 4, data is shown as mean ± SD. *P < 0.05, **P < 0.01. Unpaired *t*-test. Percentage of human nuclei (**E**) and total murine nuclei (**F**) in sequential sections of male DUX4-^APN+^MMSCs grafts, treated as indicated. Each data point is the mean of four different sections/murine TA encompassing the engraftment site (according to the scheme in Fig. S4D). Animals were sacrificed 4 weeks following transplantation. Data is a pool of two independent experiments, *n* ≧ 3 mice/group. Data is shown as mean ± SD, **P < 0.01, One-way ANOVA with Dunnett’s multiple comparisons test. **G** Human DNA quantification by qPCR in TRIzol DNA-fractions of male DUX4-^APN+^MMSCs grafts, treated as indicated, and sacrificed after 4 weeks. All samples were analysed using human TITIN gene and normalized to murine PTGER. Data is from one experiment, *n* = 4. Data is shown as mean ± SD, *P < 0.05, One-way ANOVA with Dunnett’s multiple comparisons test.

DUX4 impairs muscle regeneration through multiple processes, including cell death [6]. To assess DUX4 toxicity, we monitored mCherry-expressing human cells in vivo (Fig. S4A). Since a previous model suggests that DUX4 toxicity depends on the sex [62], we transplanted both female and male animals. Female mice were ovariectomized and, after 15 days, transplanted with DUX4- and CTR-^APN+^MMSCs and treated with E_2_ or vehicle according to the previous schedule (see Fig. 3A). Time-course imaging of mCherry indicated a gradual decrease of the signal in both DUX4- and CTR-^APN+^MMSCs transplanted animals (Fig. 4A, B). DUX4-^APN+^MMSCs transplanted muscles showed a substantial reduction of mCherry compared to CTR-^APN+^MMSCs during the early phase of muscle regeneration, suggesting noticeable DUX4-mediated cell death (compare Fig. 4A to B). E_2_ restored the signal in DUX4-transplanted mice (Fig. 4A), whereas it was ineffective in CTR-transplanted ones (Fig. 4B). Molecular analysis confirmed reduced mCherry protein levels in DUX4-compared to CTR-^APN+^MMSCs muscles and recovery after E_2_ only in DUX4-transplanted samples (Fig. 4C), supporting imaging data. The discrepancy in the mCherry signal between western blot (WB) and immunofluorescence (IMF) is likely due to the imaging detection limit of mCherry progressively diffusing along the mixed myofiber (Fig. S4B). Similarly, 3β-diol restored mCherry protein levels in male DUX4-grafts (Fig. 4D), while no effects were observed in CTR-ones (Fig. S4C). Thus, estrogen counteracts DUX4 toxicity in both sexes.

To further quantify estrogen recovery activity, we considered the number of human nuclei (Lamin AC^+^) in transversal sections encompassing the engraftment site (Fig S4D, E) in an independent group of animals. Indeed, this number was increased by E_2_ and 3β-diol (Fig. 4E), with the 3β-diol showing a more potent recovery action, both considering the total number of human nuclei and the intramyofiber ones (Figs. 4E and S4F). As control, the number of murine nuclei in the same sections was not altered by hormones (Fig. 4F). The increased number of human nuclei in hormone-treated DUX4-^APN+^MMSCs grafts correlated with an increased number of human nuclei in murine myofibers (Fig. S4F). To exclude sampling bias in the random choice of muscle sections, the quantification of human DNA content in the contralateral engrafted muscles was increased by E_2_ and 3β-diol, both if measured as levels relative to murine DNA (Fig. 4G) or as absolute human DNA content (Fig. S4G). Similar data were observed in DUX4-ImMyobs muscles, although not reaching statistical significance, probably due to the low number of engrafted human cells (Fig. S4H, I).

These data indicate that estrogen antagonizes DUX4-dependent cell death. The lack of hormone activity in CTR-^APN+^MMSCs and murine nuclei excludes a pro-proliferative function of estrogen, at least at these time points, confirming the specific activity towards DUX4.

### Estrogen rescues DUX4-impaired muscle regeneration and running functionality

**Fig. 5:**
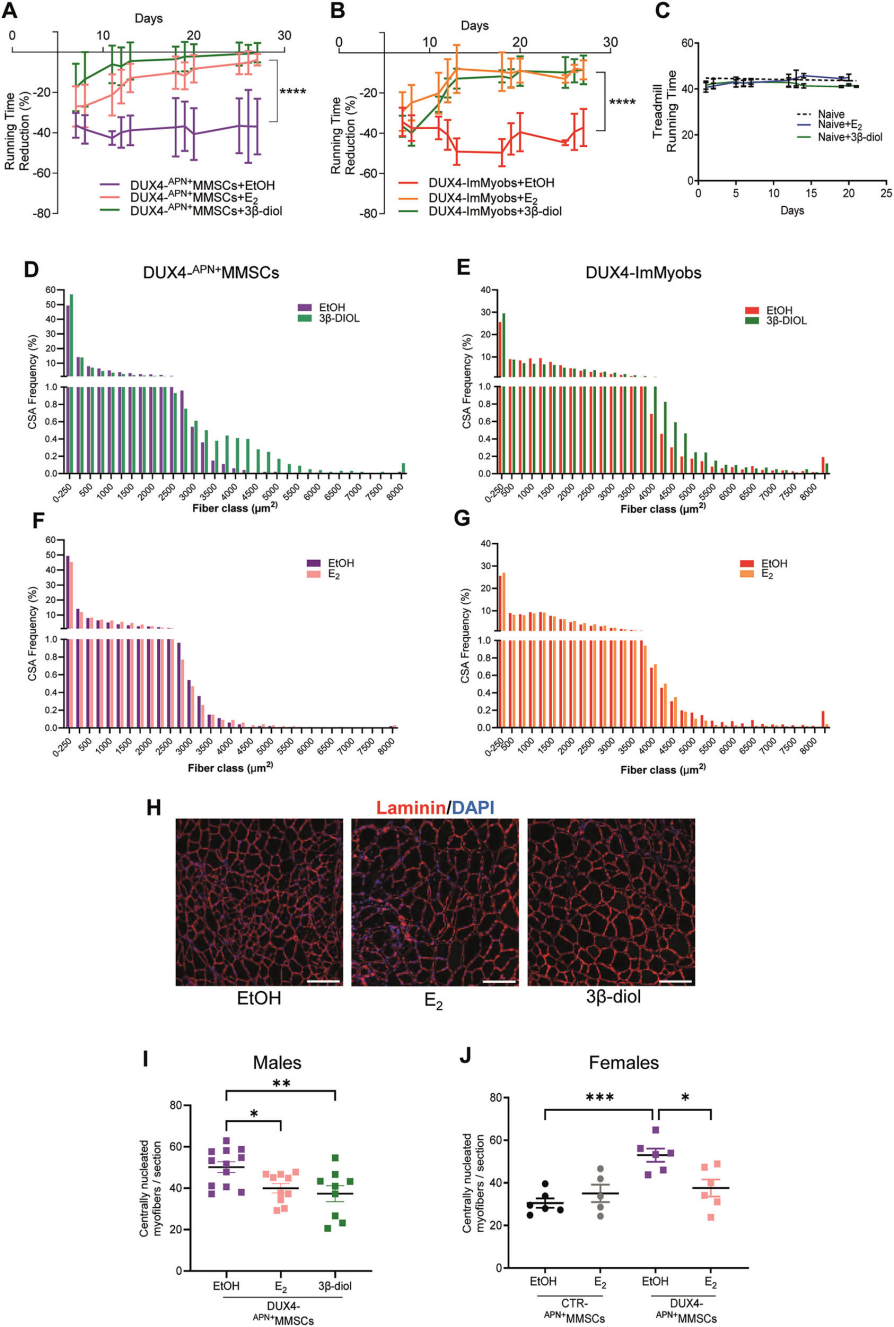
Estrogen rescues DUX4-impaired muscle running functionality and regeneration. Summary of treadmill test data performed with male mice treated as indicated. The reduction was calculated relative to the mean of a 3-day basal-run test for each mouse. Data is a pool of two (**A**) or one experiment (**B**). DUX4-^APN+^MMSCs: *n*_EtOH_ = 6, *n*_E2_ = 10, *n*_3β-diol_ = 7. DUX4-ImMyobs: *n*_EtOH_ = 4, *n*_E2_ = 4, *n*_3β-diol_ = 6. Data is shown as mean ± SD. ****P < 0.0001, mixed effects model ANOVA (**A**, hormone treatment factor *p* < 0.0001, matching *p* < 0.0001; **B**, hormone treatment factor *p* < 0.0001, matching *p* < 0.0001, no equal variability assumption). **C** Treadmill data of healthy mice treated as indicated. Data is a pool of two experiments, *n*_EtOH_ = 21, *n*_E2_ = 5, *n*_3β-diol_ = 4. CSA distribution in the 10–8000 µm^2^ range of male mice transplanted with DUX4-^APN+^MMSCs (**D**–**F**) or DUX4-ImMyobs (**E**–**G**) and treated as indicated. Each bar is the mean of 4 mice. Four sections/mouse were evaluated using the MyoEngraftmentProfiler” pipeline (Fig. S6, see STAR Methods section). Data is a pool of two experiments. *P* < 0.05 Kolmogorov-Smirnov test. **H** Representative images of male DUX4-^APN+^MMSCs graft sections, treated as indicated. Animals were sacrificed 4 weeks following transplantation. Laminin stains myofiber (red) and DAPI counterstains nuclei (blue). Scale bar is 50 µm. Quantification of CNF (centrally nucleated myofibers in transversal sections, containing at least one central nucleus in TA muscle of male DUX4-^APN+^MMSCs (**I**) or female CTR- or DUX4-^APN+^MMSCs (**J**) grafts. Animals were sacrificed five (**I**) or 4 weeks (**J**) after transplantation. Data in **I** is a pool of three independent experiments. Data in **J** is from one experiment. *n* = 4 mice/group, 3 sections/mouse (**I**). *n* = 3 mice, 2 sections/mouse (**J**), Data is shown as mean ± SD. *P < 0.05, **P < 0.01, one-way ANOVA with Dunnett’s multiple comparisons test.

Given the beneficial activity of estrogens, we investigated the hormone effect on DUX4-mediated functional impairment. E_2_ and 3β-diol strongly improved the running ability of DUX4-^APN+^MMSCs (Fig. 5A) and DUX4-ImMyobs (Fig. 5B) transplanted mice. ImMyobs showed a delayed recovery compared to ^APN+^MMSCs, possibly due to their lower engraftment levels (see Fig. 2A). No changes in animal weight (Fig. S5A) or in the running ability of healthy mice (Fig. 5C) were observed, excluding a trophic function of the hormones.

The cross-sectional area (CSA) of myofibers, a feature of newly formed myofibers and a readout of muscle size, was increased by 3β-diol, accompanied by an increased percentage of myofibers with high CSA values (range: 3000–6000 µm²) (Fig. 5D, H). Conversely, 3β-diol was ineffective in CTR- ^APN+^MMSCs (Fig. S5B). DUX4-ImMyob showed a similar tendency although without reaching statistical significance (Fig. 5E). Unexpectedly, E_2_ was ineffective on the CSA in both ^APN+^MMSCs and ImMyobs grafts (Fig. 5F, G). Since E_2_ improved the running ability of DUX4-engrafted animals (Fig. 5A, B), this data suggests that such improvement is minimally attributable to the increased cross-sectional area of the myofibers.

We considered another parameter changing during muscle regeneration: the number of centrally nucleated myofibers (CNFs), a readout of the completeness of regenerating myofibers. This value was increased in muscles engrafted with DUX4- compared to CTR-^APN+^MMSCs (Fig. 5J), indicating that DUX4 is associated with reduced myofiber completeness. Differently from CSA, both hormones significantly reduced CNF, independently of animal sex (Fig. 5J, I), whereas they were ineffective in CTR-^APN+^MMSCs muscles (Figs. 5J and S5C).

**Fig. 6:**
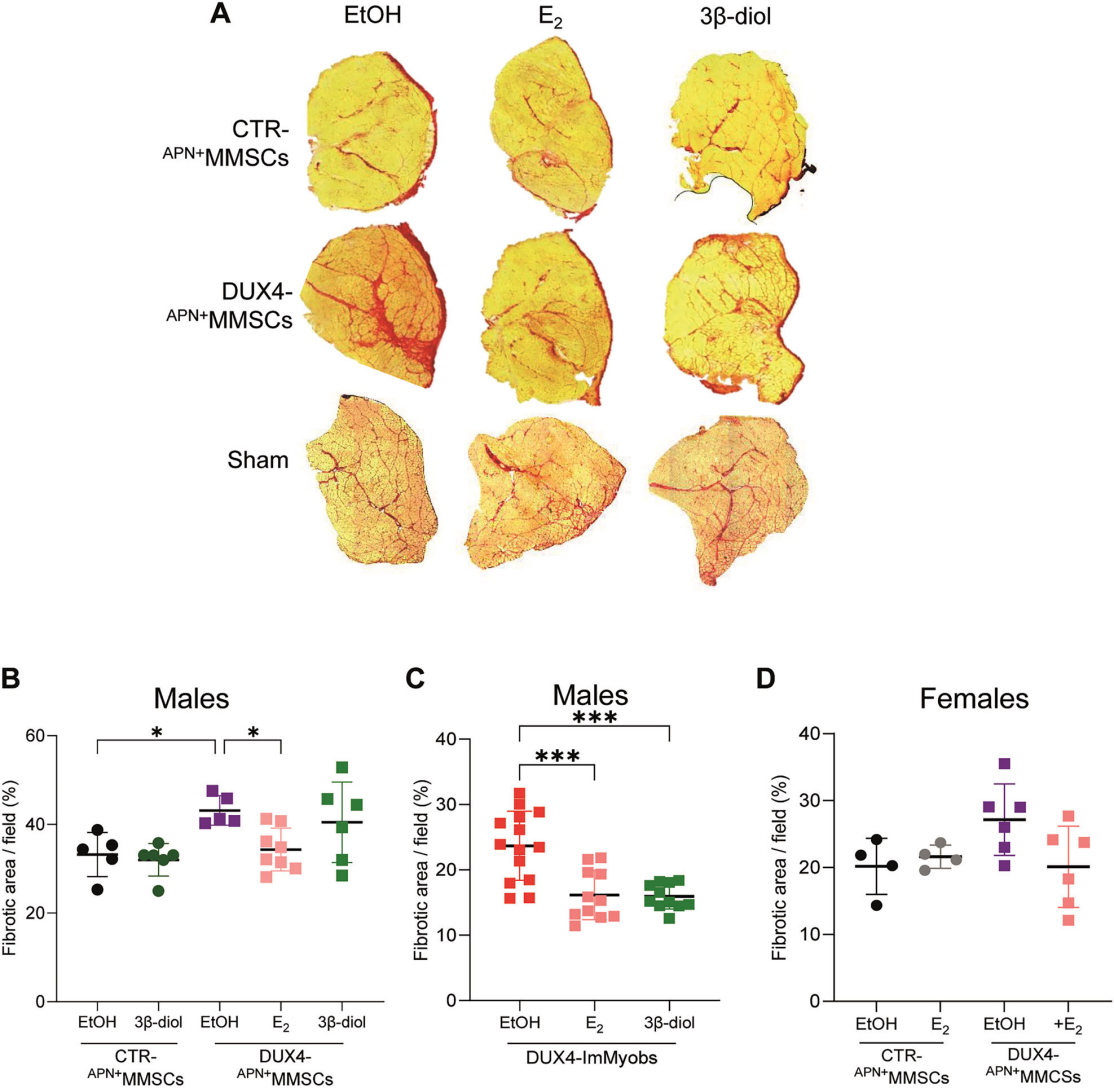
Estrogen rescues DUX4-induced muscle fibrosis. **A** Representative images of muscle fibrosis in transversal sections of CTR- or DUX4-^APN+^MMSCs or matrigel (Sham) grafts and treated as indicated. Mice were sacrificed 4 weeks (^APN+^MMSCs) or 35 days (Sham) following transplantation. Images were obtained by picrosirius red staining. Quantification of muscle fibrosis in transversal sections of male (**B**, **C**) or female (**D**) grafts transplanted with indicated cells and treated as indicated. Data is expressed as a percentage of red picrosirius^+^ pixels to the total (red+yellow) pixel number. Data is from one CTR-^APN+^MMSCs or a pool of two DUX4-^APN+^MMSCs independent experiments (**B**, **C**) or one experimental group in **D**. Animals in **B**, **C** were sacrificed 4 weeks after transplantation, those in **D** 5 weeks after transplantation. *n*_CTR-APN+MMSCs_ = 3 mice/group, 2 sections/mouse, *n*_DUX4-APN+MMSCs_≥3mice/group, 2 sections/mouse. *n*_DUX4-ImMyobs_ = 4 mice/group, three sections/mouse. Data is shown as mean ± SD. *P < 0.05, ***P < 0.001. one-way ANOVA with Dunnett’s multiple comparisons test.

One prominent feature of DUX4-induced muscle degeneration is also increased fibrosis in humans and murine muscles [24, 63]. Indeed, the engraftment of DUX4-expressing human cells was associated with enhanced muscle fibrosis compared to CTR cells and the sham group (Fig. 6A, B, D). E_2_ and 3β-diol reduced such fibrosis in both DUX4-^APN+^MMSCs and DUX4-ImMyobs engrafts (Fig. 6B, C), whereas they were ineffective in the CTR and sham groups (Figs. 6B, S5D). A similar trend was observed in the female groups, although it did not reach statistical significance (Fig. 6D). DUX4-ImMyobs were associated with decreased fibrosis compared to DUX4-^APN+^MMSCs (compare Fig. 6B and C), likely due to the higher number of engrafted ^APN+^MMSCs cells compared to ImMyobs (see Fig. 2A). Engraftment of CTR-^APN+^MMSCs was associated with reduced muscle fibrosis compared to the sham group (Fig. 6A), supporting the ability of CTR-cells to rescue running ability of CTR-cells engrafted mice (see Fig. 2D).

These data demonstrate that E_2_ and 3β-diol antagonize DUX4 activities and restore the muscle regeneration impaired by the engraftment of DUX4-expressing human cells in this heterokaryon model.

## Discussion

We employed a novel humanized mouse model of FSHD and reported the ability of the estrogens E_2_ and 3β-diol to antagonize DUX4 transcriptional activity, counteract its toxicity, and rescue the muscle regeneration and functionality impaired by this gene.

In this heterokaryon model, relatively few DUX4-expressing human cells impair the muscle regeneration of the murine TA, resembling the conditions in human FSHD muscle. The activation of murine targets by DUX4 expressed in human cells confirms the “diffusible” activity of DUX4 into nearby nuclei of the same myofiber. It supports the hypothesis that a small number of cells can be sufficient to harm the regenerative process of the entire FSHD tissue [61].

In this model, DUX4 promoted cell loss, reduced muscle regeneration, and increased fibrosis, leading to impaired muscle function. These data confirm the multiple activities of DUX4, by which it hampers the muscle regeneration process [64, 65]. Whether additional DUX4 mediators can operate on the murine muscle remains to be ascertained: a critical player may be the increased oxidative stress originated by DUX4. In fact, elevated levels of ROS trigger muscle weakness and fatigue [66, 67].

Treatment with E_2_ or 3β-diol corrected many features, including cell death. A debated issue in the field is whether hormone-beneficial activity is due to a pro-proliferative function of estrogen receptors. Three lines of evidence oppose this hypothesis: (i) ERβ is not a pro-proliferative transcription factor [68, 69]; (ii) the inhibition of DUX4 transcriptional activity by hormone treatment is evident early, preceding changes in human DNA/cell content (see Fig. 3D); (iii) in the used time frame, the CTR-^APN+^MMSCs did not show cell survival changes (see Fig. 4B, C). Therefore, we can exclude that the pro-regenerative function of estrogen is due to increased cell proliferation, and instead, it appears to be contributed by a DUX4-specific antagonism of these hormones. ERβ activity is not mediated by direct interaction with DUX4 [18]. It interferes with DUX4 nuclear localization [18], a process associated with the increased activity of this gene during the early phase of muscle differentiation. The ability of estrogen to alter intracellular localization has been reported for other factors [70, 71] and is considered a dominant response in some tumors besides a factor affecting sex-related differences [72]. Whether ERβ activity is affected by other nuclear receptors known to crosstalk with DUX4 in other tissues [73] remains an open question.

Hormones not only antagonized DUX4 toxicity but even increased cell survival (see graph in Fig. 4C). These results might be attributed to the de-differentiating and stemness function of DUX4 in the early embryonal stage and to the positive regulation of cell proliferation [74–76].

An additional beneficial action of estrogen toward cells other than MuSCs cannot be excluded. Indeed, animal models of overexpressed DUX4 and studies on muscle tissues of FSHD patients have reported an accumulation and impairment of fibroadipogenic (FAPs) precursor cells in the muscle [23, 41]. Whether estrogens can “correct” also this phenomenon by impairing a possible crosstalk between DUX4 and FAPs remains to be ascertained.

The absence of an improvement in muscle fibrosis and regeneration features in CTR-grafts or sham mice by estrogen would exclude a general beneficial effect of these hormones. However, this lack might be masked by the immunodeficient rodent model used; the NGS strain is B and T cell-deficient, has low natural killer (NK) cytotoxic activity, and is macrophage-defective. These features can hide the effects of estrogen on muscle inflammation and the consequent regenerative process. However, although numerous studies have reported a positive impact of E_2_ on musculoskeletal tissue, primarily in animal models, this remains a subject of lively debate, especially on the mechanism that may underlie this benefit [77–79].

We demonstrated anabolic activity of 3β-diol by forming large myofibers in DUX-MMSCs transplanted muscle. To the best of our knowledge, no studies have reported the effect of this hormone, probably due to the overwhelming positive activity of testosterone, from which it derives, on muscle mass and strength. Our data points to a testosterone-independent, positive effect of 3β-diol and ERβ on human muscle cells. The reason for the inefficacy of E_2_ on the same parameter remains unexplained. The recruitment of different cofactors or activation of different targets by the two ligands to ERβ can be a reasonable explanation [80].

Finally, this humanized mouse model evidenced the potential impact of hMMSC in FSHD. The stem cell niche is important during muscle regeneration, contributing to the expansion and self-renewal of skeletal muscle stem cells [81, 82]. Given the impairment of muscle regeneration in FSHD muscle, the potential contribution of hMMSCs in such alteration is arguable. The expression of DUX4 in ^APN+^MMSCs from FSHD patients confirms that DUX4 is also expressed in cells other than myoblasts when primed for muscle differentiation [50].

Overall, our results support the estrogens as a potential modifying factor in FSHD and a possible protective role towards FSHD skeletal muscle. This data encourages further studies to evaluate the effect of hormone changes or alterations in FSHD patients.

## Supplementary information

Supplementary Material

Original Data

## Data Availability

All data generated or analysed during this study are included in this published article and its supplementary information files.

## References

[1] Deenen JC, Arnts H, van der Maarel SM, Padberg GW, Verschuuren JJ, Bakker E, et al. Population-based incidence and prevalence of facioscapulohumeral dystrophy. Neurology. 2014;83:1956–9. 10.1212/WNL.0000000000000797 PMC4166358 25122204

[2] Hamel J, Tawil R. Facioscapulohumeral muscular dystrophy: update on pathogenesis and future treatments. Neurotherapeutics. 2018;15:863–71. 30361930 10.1007/s13311-018-00675-3 PMC6277282

[3] Daxinger L, Tapscott SJ, van der Maarel SM. Genetic and epigenetic contributors to FSHD. Curr Opin Genet Dev. 2015;33:56–61. 26356006 10.1016/j.gde.2015.08.007 PMC4674299

[4] Lemmers RJ, van der Vliet PJ, Klooster R, Sacconi S, Camano P, Dauwerse JG, et al. A unifying genetic model for facioscapulohumeral muscular dystrophy. Science. 2010;329:1650–3. 20724583 10.1126/science.1189044 PMC4677822

[5] Gabriëls J, Beckers MC, Ding H, De Vriese A, Plaisance S, van der Maarel SM, et al. Nucleotide sequence of the partially deleted D4Z4 locus in a patient with FSHD identifies a putative gene within each 3.3 kb element. Gene. 1999;236:25–32. 10433963 10.1016/s0378-1119(99)00267-x

[6] Banerji CRS, Zammit PS. Pathomechanisms and biomarkers in facioscapulohumeral muscular dystrophy: roles of DUX4 and PAX7. EMBO Mol Med. 2021;13:e13695. 34151531 10.15252/emmm.202013695 PMC8350899

[7] Tawil R, Forrester J, Griggs RC, Mendell J, Kissel J, McDermott M, et al. Evidence for anticipation and association of deletion size with severity in facioscapulohumeral muscular dystrophy. The FSH-DY Group. Ann Neurol. 1996;39:744–8. 8651646 10.1002/ana.410390610

[8] Wohlgemuth M, Lemmers RJ, Jonker M, van der Kooi E, Horlings CG, van Engelen BG, et al. A family-based study into penetrance in facioscapulohumeral muscular dystrophy type 1. Neurology. 2018;91:e444. 29997197 10.1212/WNL.0000000000005915 PMC6093768

[9] Padberg GW, van Engelen BGM, Voermans NC. Facioscapulohumeral Disease as a myodevelopmental disease: applying Ockham’s razor to its various features. J Neuromuscul Dis. 2023;10:15–27. 36872787 10.3233/JND-221624

[10] Deenen JCW, Horlings CGC, Voermans NC, van Deutekom JPA, Faber CG, van Kooi AJ, et al. Population-based incidence rates of 15 neuromuscular disorders: a nationwide capture-recapture study in the Netherlands. Neuromuscul Disord. 2024;42:9–18. 10.1016/j.nmd.2024.07.006 39116821

[11] Deenen JC, van Deutekom JPA, Faber CG, van Kooi AJ, Kuks JB, Notermans NC, et al. The epidemiology of neuromuscular disorders: age at onset and gender in the Netherlands. Neuromuscul Disord. 2016;26:6–12. 10.1016/j.nmd.2016.04.011 27212207

[12] Zatz M, Marie SK, Cerqueira A, Vainzof M, Pavanello RC, Passos-Bueno MR. The facioscapulohumeral muscular dystrophy (FSHD1) gene affects males more severely and more frequently than females. Am J Med Genet. 1998;77:155–61. 9605290

[13] Tonini MM, Pavanello RC, Gurgel-Giannetti J, Lemmers RJ, van der Maarel SM, Frants RR, et al. Homozygosity for autosomal dominant facioscapulohumeral muscular dystrophy (FSHD) does not result in a more severe phenotype. J Med Genet. 2004;41:e17. 14757867 10.1136/jmg.2003.010637 PMC1735661

[14] Sakellariou P, Kekou K, Fryssira H, Sofocleous C, Manta P, Panousopoulou A, et al. Mutation spectrum and phenotypic manifestation in FSHD Greek patients. Neuromuscul Disord. 2012;22:11–5. 10.1016/j.nmd.2011.11.001 22357364

[15] Ricci G, Scionti I, Sera F, Govi M, D’Amico R, Frambolli I, et al. Large scale genotype-phenotype analyses indicate that novel prognostic tools are required for families with facioscapulohumeral muscular dystrophy. Brain. 2013;136:3408–17. 24030947 10.1093/brain/awt226 PMC3808686

[16] Ruggiero L, Mele F, Manganelli F, Bruzzese D, Ricci G, Vercelli L, et al. Phenotypic variability among patients with D4Z4 reduced allele facioscapulohumeral muscular dystrophy. JAMA Netw Open. 2020;3:e204040. 32356886 10.1001/jamanetworkopen.2020.4040 PMC7195625

[17] Lin F, Wang ZQ, Lin MT, Murong SX, Wang N. New insights into genotype-phenotype correlations in Chinese facioscapulohumeral muscular dystrophy: a retrospective analysis of 178 patients. Chin Med J. 2015;128:1707–13. 26112708 10.4103/0366-6999.159336 PMC4733718

[18] Teveroni E, Pellegrino M, Sacconi S, Calandra P, Cascino I, Farioli-Vecchioli S, et al. Estrogens enhance myoblast differentiation in facioscapulohumeral muscular dystrophy by antagonizing DUX4 activity. J Clin Investig. 2017;127:1531–45. 28263188 10.1172/JCI89401 PMC5373881

[19] Stubgen JP, Stipp A. Facioscapulohumeral muscular dystrophy: a prospective study of weakness and functional impairment. J Neurol. 2010;257:1457–64. 20352247 10.1007/s00415-010-5544-1

[20] Mul K, Horlings CGC, Voermans NC, Schreuder THA, van Engelen BGM. Lifetime endogenous estrogen exposure and disease severity in female patients with facioscapulohumeral muscular dystrophy. Neuromuscul Disord. 2018;28:508–11. 29655530 10.1016/j.nmd.2018.02.012

[21] Saad NY, Al-Kharsan M, Garwick-Coppens SE, Chermahini GA, Harper MA, Palo A, et al. Human miRNA miR-675 inhibits DUX4 expression and may be exploited as a potential treatment for Facioscapulohumeral muscular dystrophy. Nat Commun. 2021;12:7128. 34880230 10.1038/s41467-021-27430-1 PMC8654987

[22] Krom YD, Thijssen PE, Young JM, den Hamer B, Balog J, Yao Z, et al. Intrinsic epigenetic regulation of the D4Z4 macrosatellite repeat in a transgenic mouse model for FSHD. PLoS Genet. 2013;9:e1003415. 23593020 10.1371/journal.pgen.1003415 PMC3616921

[23] Dandapat A, Bosnakovski D, Hartweck LM, Arpke RW, Baltgalvis KA, Vang D, et al. Dominant lethal pathologies in male mice engineered to contain an X-linked DUX4 transgene. Cell Rep. 2014;8:1484–96. 25176645 10.1016/j.celrep.2014.07.056 PMC4188423

[24] Bosnakovski D, Shams AS, Yuan C, da Silva MT, Ener ET, Baumann CW, et al. Transcriptional and cytopathological hallmarks of FSHD in chronic DUX4-expressing mice. J Clin Investig. 2020;130:2440–55. 10.1172/JCI133303 PMC7190912 32250341

[25] Jones TI, Chew GL, Barraza-Flores P, Schreier S, Ramirez M, Wuebbles RD, et al. Transgenic mice expressing tunable levels of DUX4 develop characteristic facioscapulohumeral muscular dystrophy-like pathophysiology ranging in severity. Skelet Muscle. 2020;10:28. 32278354 10.1186/s13395-020-00227-4 PMC7149937

[26] Giesige CR, Wallace LM, Heller KN, Eidahl JO, Saad NY, Fowler AM, et al. AAV-mediated follistatin gene therapy improves functional outcomes in the TIC-DUX4 mouse model of FSHD. JCI Insight. 2018;3:e123031. 10.1172/jci.insight.123538 PMC6302942 30429376

[27] Wallace LM, Garwick SE, Mei W, Belayew A, Coppee F, Ladner KJ, et al. DUX4, a candidate gene for facioscapulohumeral muscular dystrophy, causes p53-dependent myopathy in vivo. Ann Neurol. 2011;69:540–52. 21446026 10.1002/ana.22275 PMC4098764

[28] Snider L, Geng LN, Lemmers RJ, Kyba M, Ware CB, Nelson AM, et al. Facioscapulohumeral dystrophy: incomplete suppression of a retrotransposed gene. PLoS Genet. 2010;6:e1001181. 21060811 10.1371/journal.pgen.1001181 PMC2965761

[29] Beermann ML, Homma S, Miller JB. Proximity ligation assay to detect DUX4 protein in FSHD1 muscle: a pilot study. BMC Res Notes. 2022;15:6. 35538497 10.1186/s13104-022-06054-8 PMC9092897

[30] Murphy K, Zhang A, Bittel AJ, Chen YW. Molecular and phenotypic changes in FLExDUX4 mice. J Pers Med. 2023;13:20. 10.3390/jpm13071040 PMC10381554 37511653

[31] Kuiper GG, Carlsson B, Grandien K, Enmark E, Häggblad J, Nilsson S, et al. Comparison of the ligand binding specificity and transcript tissue distribution of estrogen receptors alpha and beta. Endocrinology. 1997;138:8–17. 10.1210/endo.138.3.4979 9048584

[32] Tanabe H, Mutai H, Sasayama D, Sasamoto H, Miyashiro Y, Sugiyama N, et al. Sex differences in serum levels of 5α-androstane-3β, 17β-diol, and androstenediol in the young adults: a liquid chromatography-tandem mass spectrometry study. PLoS ONE. 2021;16:e0261386. 34910781 10.1371/journal.pone.0261440 PMC8673626

[33] Heldring N, Pike A, Andersson S, Matthews J, Cheng G, Hartman J, et al. Estrogen receptors: how do they signal and what are their targets. Physiol Rev. 2007;87:905–31. 17615392 10.1152/physrev.00026.2006

[34] Wosczyna MN, Rando TA. A muscle stem cell support group: coordinated cellular responses in muscle regeneration. Dev Cell. 2018;46:135–42. 30016618 10.1016/j.devcel.2018.06.018 PMC6075730

[35] Giordani L, He GJ, Negroni E, Sakai H, Law JYC, Siu MM, et al. High-dimensional single-cell cartography reveals novel skeletal muscle-resident cell populations. Mol Cell. 2019;74:595–608.e11. 10.1016/j.molcel.2019.02.026 30922843

[36] Dellavalle A, Sampaolesi M, Tonlorenzi R, Tagliafico E, Sacchetti B, Perani L, et al. Pericytes of human skeletal muscle are myogenic precursors distinct from satellite cells. Nat Cell Biol. 2007;9:255–67. 17293855 10.1038/ncb1542

[37] Dellavalle A, Maroli G, Covarello D, Azzoni E, Innocenzi A, Perani L, et al. Pericytes resident in postnatal skeletal muscle differentiate into muscle fibres and generate satellite cells. Nat Commun. 2011;2:370. 21988915 10.1038/ncomms1508

[38] Lorant J, Saury C, Schleder C, Robriquet F, Lieubeau B, Négroni E, et al. Skeletal muscle regenerative potential of human mustem cells following transplantation into injured mice muscle. Mol Ther. 2018;26:454–67. 10.1016/j.ymthe.2017.10.013 PMC5835152 29221805

[39] Costantini M, Testa S, Fornetti E, Fuoco C, Sanchez RC, Nie M, et al. Biofabricating murine and human myo-substitutes for rapid volumetric muscle loss restoration. EMBO Mol Med. 2021;13:e13268. 10.15252/emmm.202012778 PMC7933978 33587336

[40] Genovese P, Patel A, Ziemkiewicz N, Paoli A, Bruns J, Case N, et al. Co-delivery of fibrin-laminin hydrogel with mesenchymal stem cell spheroids supports skeletal muscle regeneration following trauma. J Tissue Eng Regen Med. 2021;15:e01495. 10.1002/term.3243 PMC8648985 34551191

[41] Di Pierro L, Giacalone F, Ragozzino E, Saccone V, Tiberio F, De Biasi M, et al. Non-myogenic mesenchymal cells contribute to muscle degeneration in facioscapulohumeral muscular dystrophy patients. Cell Death Dis. 2022;13:779. 36114172 10.1038/s41419-022-05233-6 PMC9481542

[42] Statland JM, Shah B, Henderson D, Van Der Maarel S, Tapscott SJ, Tawil R. Muscle pathology grade for facioscapulohumeral muscular dystrophy biopsies. Muscle Nerve. 2015;52:521–6. 25704033 10.1002/mus.24621 PMC4546927

[43] Statland JM, Odrzywolski KJ, Shah B, Henderson D, Fricke AF, van der Maarel SM, et al. Immunohistochemical characterization of facioscapulohumeral muscular dystrophy muscle biopsies. J Neuromuscul Dis. 2015;2:269–80. 26345300 10.3233/JND-150077 PMC4560242

[44] van Splunder H, Villacampa P, Martínez-Romero A, Graupera M. Pericytes in the disease spotlight. Trends Cell Biol. 2024;34:14–26. 10.1016/j.tcb.2023.06.001 PMC10777571 37474376

[45] Tedesco FS, Moyle LA, Perdiguero E. Muscle interstitial cells: a brief field guide to non-satellite cell populations in skeletal muscle. Methods Mol Biol. 2017;1556:15–28. 10.1007/978-1-4939-6771-1_7 28247348

[46] Sacchetti B, Funari A, Remoli C, Giannicola G, Kogler G, Liedtke S, et al. No identical “mesenchymal stem cells” at different times and sites: human committed progenitors of distinct origin and differentiation potential are incorporated as adventitial cells in microvessels. Stem Cell Rep. 2016;6:897–913. 10.1016/j.stemcr.2016.05.011 PMC4912436 27304917

[47] Cathery W, Faulkner A, Maselli D, Madeddu P. Concise review: the regenerative journey of pericytes toward clinical translation. Stem Cells. 2018;36:1295–310. 29732653 10.1002/stem.2846 PMC6175115

[48] Contreras O, Rossi FMV, Theret M. Origins, potency, and heterogeneity of skeletal muscle fibro-adipogenic progenitors-time for new definitions. Skelet Muscle. 2021;11:16. 34210364 10.1186/s13395-021-00265-6 PMC8247239

[49] Krom YD, Dumonceaux J, Mamchaoui K, den Hamer B, Mariot V, Negroni E, et al. Generation of isogenic D4Z4 contracted and noncontracted immortal muscle cell clones from a mosaic patient: a cellular model for FSHD. Am J Pathol. 2012;181:1387–401. 22871573 10.1016/j.ajpath.2012.07.007 PMC3463638

[50] Himeda CL, Debarnot C, Homma S, Beermann ML, Miller JB, Jones PL, et al. Myogenic enhancers regulate expression of the facioscapulohumeral muscular dystrophy-associated DUX4 gene. Mol Cell Biol. 2014;34:2076–87. 10.1128/MCB.00149-14 PMC4019064 24636994

[51] Bosnakovski D, Daughters RS, Xu Z, Slack JM, Kyba M. Biphasic myopathic phenotype of mouse DUX, an ORF within conserved FSHD-related repeats. PLoS ONE. 2009;4:e7003. 19756142 10.1371/journal.pone.0007003 PMC2737622

[52] Mitsuhashi H, Mitsuhashi S, Lynn-Jones T, Kawahara G, Kunkel LM. Expression of DUX4 in zebrafish development recapitulates facioscapulohumeral muscular dystrophy. Hum Mol Genet. 2013;22:568–77. 23108159 10.1093/hmg/dds467 PMC3606007

[53] Bosnakovski D, Gearhart MD, Toso EA, Ener ET, Choi SH, Kyba M. Low level DUX4 expression disrupts myogenesis through deregulation of myogenic gene expression. Sci Rep. 2018;8:1170. 30446688 10.1038/s41598-018-35150-8 PMC6240038

[54] Kesireddy V. Evaluation of adipose-derived stem cells for tissue-engineered muscle repair construct-mediated repair of a murine model of volumetric muscle loss injury. Int J Nanomed. 2016;11:1461–73. 10.2147/IJN.S101955 PMC4833361 27114706

[55] Machingal MA, Corona BT, Walters TJ, Kesireddy V, Koval CN, Dannahower A, et al. A tissue-engineered muscle repair construct for functional restoration of an irrecoverable muscle injury in a murine model. Tissue Eng Part A. 2011;17:2291–303. 21548710 10.1089/ten.tea.2010.0682 PMC3161107

[56] Ferreboeuf M, Mariot V, Bessieres B, Vasiljevic A, Attie-Bitach T, Collardeau S, et al. DUX4 and DUX4 downstream target genes are expressed in fetal FSHD muscles. Hum Mol Genet. 2014;23:171–81. 23966205 10.1093/hmg/ddt409

[57] Knopp P, Krom YD, Banerji CR, Panamarova M, Moyle LA, den Hamer B, et al. DUX4 induces a transcriptome more characteristic of a less-differentiated cell state and inhibits myogenesis. J Cell Sci. 2016;129:3816–31. 27744317 10.1242/jcs.180372 PMC5087662

[58] De Micheli AJ, Laurilliard EJ, Heinke CL, Ravichandran H, Fraczek P, Soueid-Baumgarten S, et al. Single-cell analysis of the muscle stem cell hierarchy identifies heterotypic communication signals involved in skeletal muscle regeneration. Cell Rep. 2020;30:3583–95.e5. 32160558 10.1016/j.celrep.2020.02.067 PMC7091476

[59] Nilsson ME, Vandenput L, Tivesten Å, Norlén AK, Lagerquist MK, Windahl SH, et al. Measurement of a comprehensive sex steroid profile in rodent serum by high-sensitive gas chromatography-tandem mass spectrometry. Endocrinology. 2015;156:2492–502. 25856427 10.1210/en.2014-1890

[60] Wittmann BM, Sherk A, McDonnell DP. Definition of functionally important mechanistic differences among selective estrogen receptor down-regulators. Cancer Res. 2007;67:9549–60. 17909066 10.1158/0008-5472.CAN-07-1590

[61] Tassin A, Laoudj-Chenivesse D, Vanderplanck C, Barro M, Charron S, Ansseau E, et al. DUX4 expression in FSHD muscle cells: how could such a rare protein cause a myopathy?. J Cell Mol Med. 2013;17:76–89. 23206257 10.1111/j.1582-4934.2012.01647.x PMC3823138

[62] Jones T, Jones PL. A cre-inducible DUX4 transgenic mouse model for investigating facioscapulohumeral muscular dystrophy. PLoS ONE. 2018;13:e0192657. 29415061 10.1371/journal.pone.0192657 PMC5802938

[63] Ragozzino E, Bortolani S, Di PierroL, Papait A, Parolini O, Monforte M, et al. Muscle fibrosis as a prognostic biomarker in facioscapulohumeral muscular dystrophy: a retrospective cohort study. Acta Neuropathol Commun. 2023;11:165. 37849014 10.1186/s40478-023-01660-4 PMC10583430

[64] Banerji CRS, Henderson D, Tawil RN, Zammit PS. Skeletal muscle regeneration in facioscapulohumeral muscular dystrophy is correlated with pathological severity. Hum Mol Genet. 2020;29:2746–60. 32744322 10.1093/hmg/ddaa164 PMC7530526

[65] Bosnakovski D, Chan SSK, Recht OO, Hartweck LM, Gustafson CJ, Athman LL, et al. Muscle pathology from stochastic low level DUX4 expression in an FSHD mouse model. Nat Commun. 2017;8:550. 28916757 10.1038/s41467-017-00730-1 PMC5601940

[66] Barclay JK, Hansel M. Free radicals may contribute to oxidative skeletal muscle fatigue. Can J Physiol Pharmacol. 1991;69:279–84. 2054745 10.1139/y91-043

[67] Cheng AJ, Place N, Westerblad H. Molecular basis for exercise-induced fatigue: the importance of strictly controlled cellular Ca 2+ handling. Cold Spring Harb Perspect Med. 2018;8:a029710. 28432118 10.1101/cshperspect.a029710 PMC5793735

[68] Warner M, Fan X, Strom A, Wu W, Gustafsson JÅ. 25 years of ERβ: a personal journey. J Mol Endocrinol. 2021;68:R1–9. 34546964 10.1530/JME-21-0121

[69] Mal R, Magner A, David J, Datta J, Vallabhaneni M, Kassem M, et al. Estrogen Receptor Beta (ERβ): a ligand activated tumor suppressor. Front Oncol. 2020;10:587386. 33194742 10.3389/fonc.2020.587386 PMC7645238

[70] Mahmoodzadeh S, Haase H, Sporbert A, Rharass T, Panakova D, Morano I. Nuclear translocation of the cardiac L-type calcium channel C-terminus is regulated by sex and 17beta-estradiol. J Mol Cell Cardiol. 2016;97:226–34. 27266387 10.1016/j.yjmcc.2016.06.004

[71] Lucà R, di Blasio G, Gallo D, Monteleone V, Manni I, Fici L, et al. Estrogens counteract platinum-chemosensitivity by modifying the subcellular localization of MDM4. Cancers. 2019;11:1349. 31547268 10.3390/cancers11091349 PMC6770881

[72] Pinto G, Alhaiek AA, Amadi S, Qattan AT, Crawford M, Radulovic M, et al. Systematic nucleo-cytoplasmic trafficking of proteins following exposure of MCF7 breast cancer cells to estradiol. J Proteome Res. 2014;13:1112–27. 24422525 10.1021/pr4012359 PMC4261610

[73] Quintero J, Saad NY, Pagnoni SM, Jacquelin DK, Gatica LV, Harper SQ, et al. The DUX4 protein is a co-repressor of the progesterone and glucocorticoid nuclear receptors. FEBS Lett. 2022;596:2644–58. 35662006 10.1002/1873-3468.14416

[74] Whiddon JL, Langford AT, Wong CJ, Zhong JW, Tapscott SJ. Conservation and innovation in the DUX4-family gene network. Nat Genet. 2017;49:935–40. 28459454 10.1038/ng.3846 PMC5446306

[75] Mocciaro E, Runfola V, Ghezzi P, Pannese M, Gabellini D. DUX4 role in normal physiology and in FSHD muscular dystrophy. Cells. 2021;10:3322. 34943834 10.3390/cells10123322 PMC8699294

[76] Zheng D, Wondergem A, Kloet S, Willemsen I, Balog J, Tapscott SJ, et al. snRNA-seq analysis in multinucleated myogenic FSHD cells identifies heterogeneous FSHD transcriptome signatures associated with embryonic-like program activation and oxidative stress-induced apoptosis. Hum Mol Genet. 2024;33:284–98. 37934801 10.1093/hmg/ddad186 PMC10800016

[77] Chidi-Ogbolu N, Baar K. Effect of estrogen on musculoskeletal performance and injury risk. Front Physiol. 2019;9:1834. 30697162 10.3389/fphys.2018.01834 PMC6341375

[78] Velders M, Diel P. How sex hormones promote skeletal muscle regeneration. Sports Med. 2013;43:1089–100. 23888432 10.1007/s40279-013-0081-6

[79] Tiidus PM, Enns DL. Point:Counterpoint: estrogen and sex do/do not influence post-exercise indexes of muscle damage, inflammation, and repair. J Appl Physiol. 2009;106:1010–2. 18599675 10.1152/japplphysiol.90848.2008

[80] Broekema MF, Hollman DAA, Koppen A, van den Ham HJ, Melchers D, Pijnenburg D, et al. Profiling of 3696 nuclear receptor-coregulator interactions: a resource for biological and clinical discovery. Endocrinology. 2018;159:2397–407. 29718163 10.1210/en.2018-00149

[81] Rayagiri SS, Ranaldi D, Raven A, Mohamad Azhar NIF, Lefebvre O, Zammit PS, et al. Basal lamina remodeling at the skeletal muscle stem cell niche mediates stem cell self-renewal. Nat Commun. 2018;9:1075. 29540680 10.1038/s41467-018-03425-3 PMC5852002

[82] Bröhl D, Vasyutina E, Czajkowski MT, Griger J, Rassek C, Rahn HP, et al. Colonization of the satellite cell niche by skeletal muscle progenitor cells depends on Notch signals. Dev cell. 2012;23:469–81. 22940113 10.1016/j.devcel.2012.07.014

