# Supplementary material for: Estrogen rescues muscle regeneration impaired by DUX4 in a humanized xenograft mouse model
Source: Cell Death Dis. 2025 Jul 9;16(1):508. doi: 10.1038/s41419-025-07827-2 (PMC12241518; doi:10.1038/s41419-025-07827-2)
Supplement: Supplementary file 1 — Supplementary Material [file 41419_2025_7827_MOESM1_ESM.pdf]

Supplementary information

Estrogen rescues muscle regeneration impaired by DUX4 in a humanized xenograft mouse model

Silvia Maiullari[1,2,§], Giada Mele[1,2,§], Patrizia Calandra[1], Giorgia di Blasio[1,2], Sonia Valentini[1,2], Alessio Torcinaro[1], Isabella Manni[3], Emanuela Teveroni[1], Fabio Mancino[4], Luca Proietti[5], Fabio Maiullari[6,7], Maria Pesavento[1,2], Ludovica Giorgini[1,2], Sabrina Putti[1], Roberto Rizzi[6,8], Sara Bortolani[9], Ferdinando Scavizzi[1], Marcello Raspa[1], Enzo Ricci[10], Giulia Piaggio[3], Cesare Gargioli[11], Alfredo Pontecorvi[12], Siro Luvisetto[1], Massimiliano Mazzone[13,14], Giancarlo Deidda[1], Fabiola Moretti[1#]

- **Materials and methods**
- **Fig. S1 Characterization of human muscle mesenchymal stromal cells**
- **Fig. S2 Characterization of human cell engraftments**
- **Fig. S3 Transcriptional activity of DUX4 in $^{APN+}$MMSCs**
- **Fig. S4 Estrogen activity on survival of engrafted human cells**
- **Fig. S5 Estrogen activity towards engrafted murine muscle**
- **Fig. S6 "MyoEngraftmentProfiler" pipeline developed with CellProfiler software**
- **Table S1 Features of FSHD patients**
- **Table S2 Primers and probes used for qPCR**
- **Table S3 Primary and secondary antibodies used for Western blot and Immunofluorescence**

Materials and Methods

Biopsies, cell culture, transfection, infection, and treatments

Skeletal muscle biopsies from healthy subjects were sterilely isolated, soaked in medium, and processed within 24 hrs. The collection of human biopsies was approved by the Fondazione Policlinico Universitario A. Gemelli Ethical Committee (Prot. ID 1524) and obtained after informed consent from all participants. Fifteen biopsies, derived from different muscles (quadriceps, pronator quadratus, vastus medialis, gluteus), were used for the experiments. Muscular tissue was finely minced with tweezers and scissors. The minced tissue was digested with collagenase II (0.1 mg/ml, Gibco #17101015, Thermo Fisher Scientific Inc, USA) for 45' at 37°C, and the pellet was resuspended in αMEM (Gibco #12571063), 20% heat-inactivated FBS (Gibco #A5256701), sodium pyruvate (Sigma #S8636, Merck KGaA, Darmstadt, Germany), HEPES (Sigma #H0887), Pen/Strep (Sigma #P4333), Glutamax (Gibco #35050061), and then filtered through a 100 µm, 70 µm, and 40 µm cell strainer (Falcon #352360, #352350, #352340, Corning, USA). Purified cells were plated at low cell density (0.1–1 × 10^4 cells/cm^2). A subset of cells from each biopsy at the first passage was characterized by ALP staining using the NBT/BCIP assay (Sigma #72091). Any batch of cells with less than 80% positive ALP staining was not used for further experiments. MMSCs were differentiated in DMEM/F12 medium (Gibco #11320033) with 1% horse serum (Gibco #16050122). For hormone treatment, the cells were grown in DMEM without phenol red (Gibco #31053) supplemented with glutamine, hEGF (25ng/ml, Sigma #SRP3027), 10% charcoal/dextran treated heat-inactivated FBS (Gibco #12676029), Gentamicin (Sigma # G1397), and Amphotericin B (Sigma #A2942), and differentiated in DMEM/F12 without phenol red (Gibco #21041025) supplemented with 1% charcoal-treated horse serum. MMSCs were infected with a tetracycline-inducible gene expression lentiviral vector (Lenti-X Tet-One Inducible Expression System-Puro) (Takara, Takara Bio Inc., Japan), coding for HADUX4 (MOI 0.5) + pLV-mCHerry, and were grown in αMEM medium with 20%

doxycycline-free TET-FBS serum (Takara). HADUX4 was induced by doxycycline (in vitro dose: 1μg/ml).

Lenti-X-Tet-One-HADUX was obtained by cloning HADUX in the empty vector using EcoRI and AgeI restriction enzymes, and the construct was checked by sequencing. The 5' end was constituted by the EcoRI-KozaK (CCACC)-HA-DUX4 sequence devoid of the ATG. It used the SV40 poly(A) from the vector. pLV-mCherry was a gift from Pantelis Tsoulfas (Addgene plasmid #36084; http://n2t.net/addgene:36084; RRID: Addgene_36084, Addgene, USA).

Myoblasts were grown in SkGM-2 medium (Lonza #CC-3245, Lonza, Switzerland) and differentiated in DMEM/F-12 supplemented with 1% horse serum. Primary myoblasts from healthy control subjects 03U and 12U were obtained from C. Emerson at the University of Massachusetts Medical School in Worcester, Massachusetts, USA. Male and female myoblasts were purchased from Lonza and subsequently immortalized by retroviral transduction of hTERT and CDK4-R24C (1). Immortalized myoblasts were infected with the same lentiviral vectors coding for HADUX4 and mCherry and grown in SkGM-2 medium with 10% doxycycline-free heat-inactivated TET-FBS serum (Clontech, Takara).

Each mix of human MMSCs or ImMyobs infected with Tet-DUX4 was assessed for the expression of DUX4 targets by RT-qPCR and for DUX4 protein levels before transplantation.

The identity of MCF7 cells (human breast adenocarcinoma) has been confirmed by the PowerPlex 18D system (Promega, Promega Corporation, USA) through the BMR Genomics service (last analysis: 12/2020). Mycoplasma-free conditions have been routinely tested using the MycoAlert kit (Lonza #LT07-318) and DAPI staining.

Mice

Animal studies obtained internal approval from IBCN-CNR and EMMA-Infrafrontiers Internal Animal Welfare Body (OPBA) and definitive ethical approval from the Ministry of Health (Protocol N°

999/2017-PR) and were conducted conforming to the institutional guidelines in compliance with Italian laws (DL N116, GU, suppl. 40, 18-2-1992) and ARRIVE guideline. NSG (NOD scid gamma, NOD.Cg-Prkdcscid Il2rgtm1Wjl/SzJ) mice were housed in individually ventilated cages (Tecniplast, Gazzada, Italy) at the European Mouse Mutant Archive (CNR-EMMA) Infrafrontier facility. All animals were housed in individually ventilated cages (Tecniplast, Gazzada, Italy) at a temperature of 20 ± 2 °C, a relative humidity of 55 ± 15%, with 12-15 air changes per hour, and a 12/12-h light/dark cycle (7 a.m.–7 p.m.). Certified dust-free wood bedding (Scobisone, Mucedola, Settimo Milanese, Milano, Italy) was provided in the cages. Mice were fed a standardized mouse diet (4RFN and Emma 23, Mucedola, Italy) and were provided chlorinated, filtered water ad libitum. Animals were tested for microorganisms every 3 months using 6- to 8-week-old B6N sentinels. Serology was performed according to the FELASA recommendations. The age of the animals at the start of the treatment ranged from 8 to 10 weeks. All female mice were ovariectomized and treated only with E_2. Male mice were treated with E_2 or 3β-diol. All experimental mice were fed an estrogen-free diet (Mucedola 4RF21TC, Mucedola, Italy). Treadmill assays were performed with male mice only.

For human cell transplantation, anesthetized mice were placed in a prone position on a heated surgery pad, and the area was sanitized with 70% alcohol before incision. Subsequently, a small incision of approximately 0.5 cm was performed, and a punch of muscle tissue was removed to create a pocket, into which 1x10^^{6} cells, enclosed in Matrigel (Corning #CLS356231, Corning, USA), were layered. After matrigel polymerization, a skin suture was performed.

Two days after the surgery, animals were randomly assigned to receive subcutaneous injections of 3,4µmol/100µl doxycycline (Sigma D5207) plus 100 µg/kg bw E_2 (Sigma #E2758), or 1mg/kg bw 3β-diol (BioSynth #W-105485, BioSynth, USA), or EtOH every other day. ICI (200mg/kg bd) (Faslodex, AstraZeneca, Italy) was administered weekly. E_2 dose was calculated based on the Nilsson study (2), and the 3β-diol dose was calculated based on its relative binding affinity to ERβ. Every single

experiment included treatment/s and vehicle and was performed using the same batch of MMSCs or ImMyobs mix infected with Cherry or Cherry/DUX4 expressing lentiviruses. The same number of animals was allocated to each treatment, whenever possible. No exclusion criteria were applied. Fluorescence emission was detected using the IVIS Lumina II CCD camera system, and the signal was analyzed with the Living Image 2.20 software package (Perkin Elmer, USA). The signal from each mouse was analysed using the same scale by subtracting the background mean at all time points. The background was collected close to the positive signal site. The fluorescent value at all time points was calculated as fold change relative to the starting value of each mouse set to 1. IVIS data collection was performed by an investigator who was blinded to the group and/or treatment. For sacrifice, mice were euthanized, and their TA muscles were immediately excised and used for molecular analysis or IHC. TA samples for molecular analysis were finely minced, aliquoted in two vials for mRNA and protein analysis, flash-frozen in liquid N_2, and stored at −80°C. The samples for IHC were cryopreserved using Tissue Tek OCT Compound Freezing Medium (Leica #14020108926, Leica Biosystems, USA), quickly frozen in liquid N_2-cooled isopentane, and stored at −80°C until processing.

Cell immunofluorescence

For immunofluorescence (IMF), cells were fixed with 80% acetone, blocked in 5% BSA, and then immunodecorated with primary antibodies (for antibody specifications, see Supplemental Table S3). For anti-PDGFRβ IMF, cells were fixed in 4% formaldehyde for 10' at RT, permeabilized with 0.05% Triton X-100 for 15' RT, and blocked with 0.5% BSA. Primary antibody binding was detected using AlexaFluor secondary antibodies (Thermo Fisher Scientific Inc., USA). Immunofluorescence images were acquired using the fluorescence microscope (Olympus AX70, Olympus, Tokyo, Japan) or the confocal microscope (Olympus FV1200). TUNEL$^+$ cells were detected with the *In situ* Cell Death

Detection Kit Fluorescein (Roche, Switzerland), according to the manufacturer's instructions, and images were acquired with the Ts2-FL fluorescence microscope (Nikon, Tokyo, Japan).

Flow Cytometry

For flow cytometry analysis, cells were resuspended in PBS with 1% BSA for 30' on ice. After washing in cold PBS, cells were incubated with primary antibodies for 2 hrs on ice with regular mixing. Cells were stained with a mix of anti-human NG2/MCSP APC-conjugated Monoclonal Antibody (R&D Systems, Biotechne, USA) for NG2 expression, and anti-human CD140a Monoclonal Antibody (BD Biosciences, Franklin Lakes, NJ, USA) for CD140a/PDGFRα expression and analyzed with FACS CANTO II flow cytometer (BD Biosciences). All experiments were compared to the isotype control antibody. The analysis was conducted using FlowJo software (BD Biosciences).

Histology

Cryosections (10 µm) were mounted on slides and air-dried before staining or storage. Sections were used for IMF or picrosirius red staining. For IMF, a 10-µm cryosection of muscle tissue was permeabilized with 0.3% Triton X-100/PBS for 1h, incubated with a blocking solution (1% normal goat serum, 5% BSA), and then incubated with primary antibody at 4 °C overnight. The next day, sections were washed twice with $PBS^{-/-}$ and incubated with the secondary antibody at room temperature for 1 h, then mounted in ProLong Gold with DAPI (Invitrogen #P36931, Thermo Fisher Scientific Inc., USA) for nuclear staining. All antibodies used are listed in the Supplemental Table S3. IMF images were captured using the Olympus AX70 camera. At least three non-consecutive sections (distanced at least 80 µm each other) around the transplantation site of each muscle from 3–4 mice per group were analyzed. Exclusion of damaged areas was performed manually.

For fibrosis analysis, cryosections of the TA muscle were fixed in Bouin Solution (Sigma #HT10132) for 1h at 56°C, rinsed with dH_2O, and stained for 1 h with picrosirius red solution (SR) (0.1% direct

red 80, 1.3% saturated picric acid). After dH_2O washing, stained sections were dehydrated by 1' ethanol washes (70%, 95%, and 100%), cleared with xylene for 5', and mounted. A series of micrographs from each muscle section was captured using a 10X objective on an Olympus BX41 and reconstituted to form a whole muscle cross-section. At least 3 complete cross-section images/muscles were processed. The percentage of fibrosis was semi-automatically scored by Image-J software as the ratio of red pixels to the sum of red and yellow pixels. For fibrosis and IHC analyses, the data were analysed by two independent operators, one of whom was blinded to the treatment. Cross-sectional area (CSA) and Lamin AC-positive nuclei were quantified from anti-Laminin/Lamin A/C and DAPI-stained TA muscle sections. Adjacent images at 10X magnification of the whole muscle section were acquired using an Olympus BX53 microscope equipped with an XM10 camera (Olympus, Tokyo, Japan). The analysis was performed using the "MyoEngraftmentProfiler" pipeline on the open-source software CellProfiler.

MyoEngraftmentProfiler pipeline

The "MyoEngraftmentProfiler" was developed using CellProfiler software (3). Specifically, we designed the pipeline to quantify the CSA of muscle fibers and their distribution, the total number of nuclei, the total number of Lamin A/C-positive nuclei, and the number of Lamin A/C-positive nuclei located within or outside muscle fibers (Fig. S6). To run and analyze the whole muscle sections, made of a large number of tiles, we increased the size of the Java heap memory on startup using the following command lines in the command prompt:

```
cd C:\Program Files\CellProfiler
set _JAVA_OPTIONS=-Xmx2g
CellProfiler.exe
```

Once CellProfiler was launched, we ran the pipeline in CellProfiler.

RNA and DNA isolation, cDNA synthesis, and qPCR

mRNA levels were evaluated by quantitative real-time PCR using a 7900HT Fast Real-Time PCR Instrument (Applied Biosystems, Thermo Fischer Scientific Inc.). RNA was isolated from snap-frozen muscles or cultured cells using TRIzol (Invitrogen #15596026). Tissue samples were lysed using a tissue homogenizer (Qiagen). 500 or 1000 ng of RNA was reverse transcribed into single-stranded cDNA using Ultrapure SMART MMLV Reverse Transcriptase (Takara) and subjected to PCR amplification using SYBR Green PCR Master Mix (Bioline) or TaqMan probes, as listed in Supplemental Table S2.

For human DNA quantification in DUX4-$^{APN+}$MMSCs and DUX4-ImMyobs engrafted muscles, after removing the TRIzol aqueous phase with RNA, the tubes containing the TRIzol interphase/organic phase were spun down at 12,000 x g for 5' at 4°C. Any remaining aqueous phase was carefully removed, and the extraction and purification of DNA from the interphase were performed as follows: 500 µL of Back Extraction Buffer (BEB, 4 M Guanidine Thiocyanate/50 mM Sodium Citrate NaCl/1 M Tris base) was added for every 1 ml of TRIzol. The aqueous phase was transferred to a clean tube, and 400 µL of ice-cold isopropanol was added. After incubation at RT for 5', the tube was centrifuged at 12,000 x g for 15' at 4°C, and the DNA pellet was washed with 70% EtOH. qPCR was carried out using SYBR Select master mix (Applied Biosystems) on a 7500 Real-Time PCR Instrument (Applied Biosystems). qPCR reaction was performed using 50ng total genomic DNA (mouse+human). Human DNA content was evaluated with primers for human TITIN gene normalized using mouse-specific primers for prostaglandin E receptor 2 (Ptger2) (4), relative to the mean value of EtOH-treated samples set to 1. The percentage of human genome content was evaluated using three different FOXP1 gene primer pairs consisting of a common forward primer and three different reverse-specific primers. The product of human- or mouse-specific FOXP1 was normalized to human+mouse FOXP1 product (Table S2). The number of human and mouse genomes

was obtained relative to a reference sample that was quantified by an absolute curve. The percentage results from human/mouse+human. The FOXP1 gene was chosen based on its high sequence homology between human and mouse. The operator was blinded to the animal treatment.

To evaluate the percentage of human genome content in CTR-MMSCs or CTR-ImMyobs (Fig. 2A), DNA was directly isolated from whole tibialis anterior following homogenization with Qiagen Tissue Lyser MM 300 and resuspended in lysis buffer (10mM Tris-HCl pH 8.2, 400 mM NaCl,2mM Na_2 EDTA) with proteinase K 20mg/ml and SDS 20%, incubated 2hrs at 50°C and o.n. at 37°C. DNA was then extracted using an adapted salting out protocol by adding RNAse treatment followed by phenol/chloroform extraction. qPCR was carried out using the three different FOXP1 gene primer pairs (Table S2). T0 input samples were obtained by mixing 1 × 10^6 proliferating $^{APN+}$MMSCs or Im-Myobs with 1 TA muscle. Primers and probes used for RNA and DNA analysis are listed in Supplemental Table S2.

Protein analysis

Tissues or cultured cells were lysed in RIPA buffer (50mM Tris–Cl, pH 7.5, 150mM NaCl, 1% Nonidet P-40, 0.5% Na deoxycholate, 0.1% SDS, 1mM EDTA), supplemented with 0.5 mM Na_3VO_4, 1mM PMSF, 5mM NaF, 10 µl/ml Protease inhibitor cocktail (Sigma#S8830). For Western blot (wb) analysis, proteins were resolved by SDS-PAGE and transferred to PVDF membranes (Millipore, Merck, Germany). Blots were blocked for 45' in 5% non-fat dry milk (Sigma). The membranes were then incubated with the antibodies listed in Supplemental Table S3, and the chemiluminescent reaction was resolved by the imaging system Alliance 2.7 (Uvitec, Cambridge, UK) and quantified by the software Alliance V_1607.

Treadmill test

Exercise experiments were performed on a five-lane motorized treadmill equipped with an electronic control unit (Treadmill Model LE8710, PanLab, Cornella (BCN), Spain) and an electric shock grid at one end of the treadmill. The operator was blinded to the group and animal treatment. Shock intensity was set at 0.4 mA. The inclination of the treadmill was set at 0°. Before the running test, mice were acclimated to the treadmill for 2 min, followed by a running session. Belt speed started at 12 m/min and after 5' increased by 1 m/min every 2 min until exhaustion, defined as the inability to maintain running speed. The limit for removal of mice from the treadmill was 5" on the shocker plate without attempting to reengage the treadmill. The time to exhaustion was automatically recorded from the beginning of the running session. Each mouse underwent various cycles of the treadmill test. Six days before surgery (day 0), mice were subjected to three running sessions (at -6, -3, and -2 days). The average time of these cycles was used to calculate the baseline performance for each mouse. On day seven post-surgery, mice were subjected to the first cycle of two running sessions (on days 7 and 8), followed by two days of rest. On day 11, mice were subjected to the second cycle of three running sessions (days 11, 12, and 13), followed by four days of rest. This cycle of three running sessions, followed by four days of rest, was repeated two times, on day 16 and day 23, respectively. All treadmill studies, but those with immortalized myoblasts, were repeated at least two times.

Statistical analyses

Statistical analysis was performed using Prism v8.0.2 and v9.0.1 (GraphPad) software. The number of replicates, corresponding statistical tests, and statistically significant differences are indicated in figure legends. Statistically significant differences are indicated (*$P \leq 0.05$; **$P \leq 0.01$; ***$P \leq 0.001$; ****$P \leq 0.0001$; ns, not significant). Data represent mean ± SD or SEM. Differences between groups were considered significant for $P \leq 0.05$.

References

1. Teveroni E, Pellegrino M, Sacconi S, Calandra P, Cascino I, Farioli-Vecchioli S, et al. Estrogens enhance myoblast differentiation in facioscapulohumeral muscular dystrophy by antagonizing DUX4 activity. Journal of Clinical Investigation. 2017;127(4):1531-45.
2. Nilsson ME, Vandenput L, Tivesten Å, Norlén AK, Lagerquist MK, Windahl SH, et al. Measurement of a Comprehensive Sex Steroid Profile in Rodent Serum by High-Sensitive Gas Chromatography-Tandem Mass Spectrometry. Endocrinology. 2015;156(7):2492-502.
3. Laghi V, Ricci V, De S, F., Torcinaro A. A User-Friendly Approach for Routine Histopathological and Morphometric Analysis of Skeletal Muscle Using CellProfiler Software. Diagnostics (Basel, Switzerland). 2022;12(3):4.
4. Alcoser SY, Kimmel DJ, Borgel SD, Carter JP, Dougherty KM, Hollingshead MG. Real-time PCR-based assay to quantify the relative amount of human and mouse tissue present in tumor xenografts. BMC Biotechnology. 2011;11:124.

Fig. S1

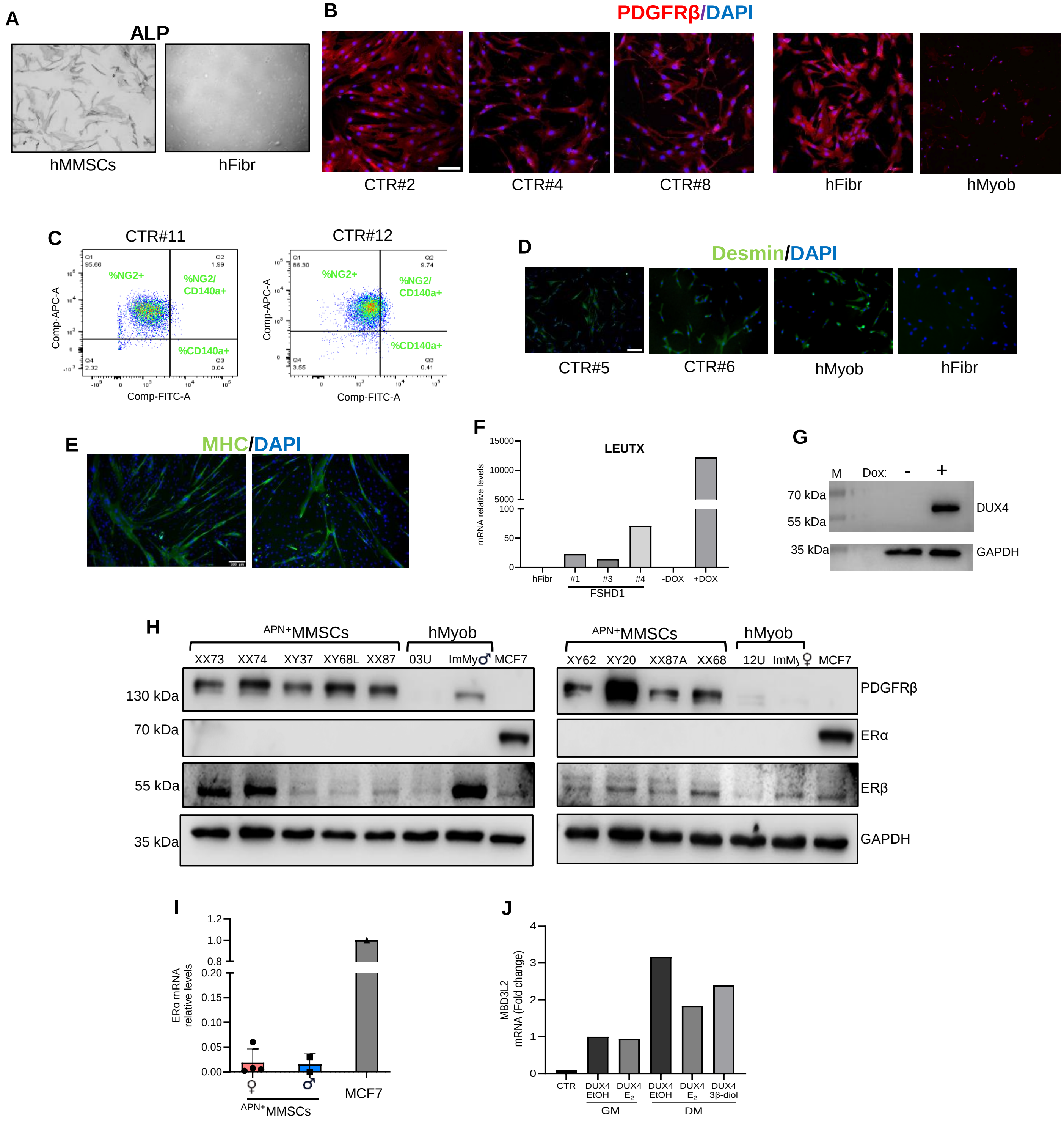

Fig. S1 Characterization of human muscle mesenchymal stromal cells. A Representative immunocytochemistry (IHC) images of alkaline phosphatase activity (ALP) in human $^{APN+}$MMSCs. Human fibroblasts (hFibr) served as a negative control. **B** Representative immunofluorescence (IMF) images of PDGFRβ (red signal) in $^{APN+}$MMSCs from healthy subjects (CTR). HFibr and human myoblasts (hMyob) served as positive and negative control, respectively. DAPI counterstains nuclei (blue). Scale bar 100µm. **C** Representative flow cytometry double staining for NG2 and CD140α (PDGFRα) in $^{APN+}$MMSCs from two healthy subjects. **D** Representative IMF images of desmin (green signal) in $^{APN+}$MMSCs. HFibr and hMyob served as positive and negative control, respectively. DAPI counterstains nuclei (blue). Scale bar 100 µm. **E** Representative IMF images of human myosin heavy chain (MHC) (green signal) in $^{APN+}$MMSCs. (MHC^{+} cells, after 11 days in differentiation medium (DM): 20±8.14, fusion index: 87.5±5.5 (mean±SD), n=3 fields/sample). DAPI counterstains nuclei (blue). Scale bar 100 µm. **F** LEUTX mRNA levels by RT-qPCR in DUX4- $^{APN+}$MMSCs -/+ Dox treatment for 48 hours and in myoblasts from 3 FSHD1 patients (#1, #3, #4) previously described (1), grown in DM for 5 days. Values are relative to the LEUTX mRNA levels in the absence of Dox, set to 1. hFibr served as negative control. **G-H** WB of indicated proteins in $^{APN+}$MMSC expressing Dox-inducible DUX4 (**G**) or from different biopsies and hMyob (**H**). (Female 03U, male 12U from Emerson's lab; ImMyob from commercial batches). MCF7 served as positive control for ERα and ERβ. **I** Human ERα mRNA levels by qRT-PCR in $^{APN+}$MMSC from male and female healthy subjects. Data are relative to levels of MCF7 set to 1. Data is shown as mean±SD. **J** human MBD3L2 mRNA levels by qRT-PCR in $^{APN+}$MMSCs transiently transfected with DUX4. Data were normalized to hAct and relative to EtOH-treated cells.

Fig. S2

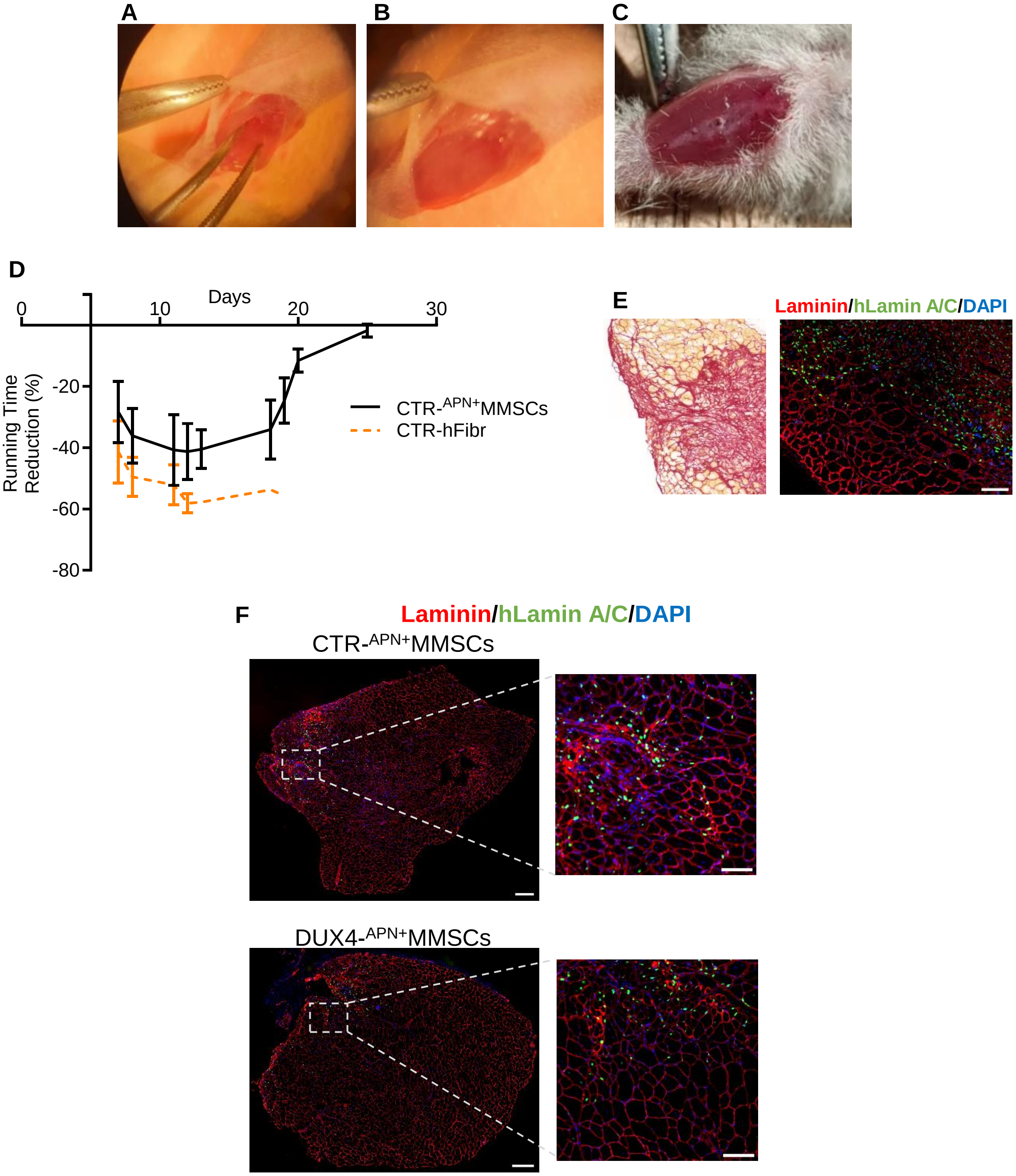

Fig. S2 Characterization of human cell engraftments. A-C Representative images of the pocket created in the TA murine muscle and the transplantation of 1 × 10^6 cells enclosed within 20µl of matrigel. **D** Treadmill data of male mice undergoing surgery and transplanted with CTR-$^{APN+}$MMSCs or human fibroblasts (CTR-hFibr). The running time reduction was calculated relative to the mean of a 3-day pre-run test for each mouse. (n=3 mice/group). Data is shown as mean±SD. **E** Representative images of Picrosirius red staining (left) and IMF (right) of CTR-hFibr grafts. Mice were sacrified two weeks following transplantation. In IMF image, laminin stains myofibers (red), human Lamin A/C stains human nuclei (green), DAPI counterstains nuclei (blue). Scale bar 100 µm. **F** Representative images of 1 section/graft from mice transplanted with CTR-$^{APN+}$MMSCs or DUX4-$^{APN+}$MMSCs. Mice were sacrified 4 weeks following transplantation. Laminin stains myofibers (red), human Lamin A/C stains human nuclei (green), DAPI counterstains nuclei (blue). Scale bar of the complete muscle section is 250 µm. Scale bar of the magnified section is 100 µm.

Fig. S3

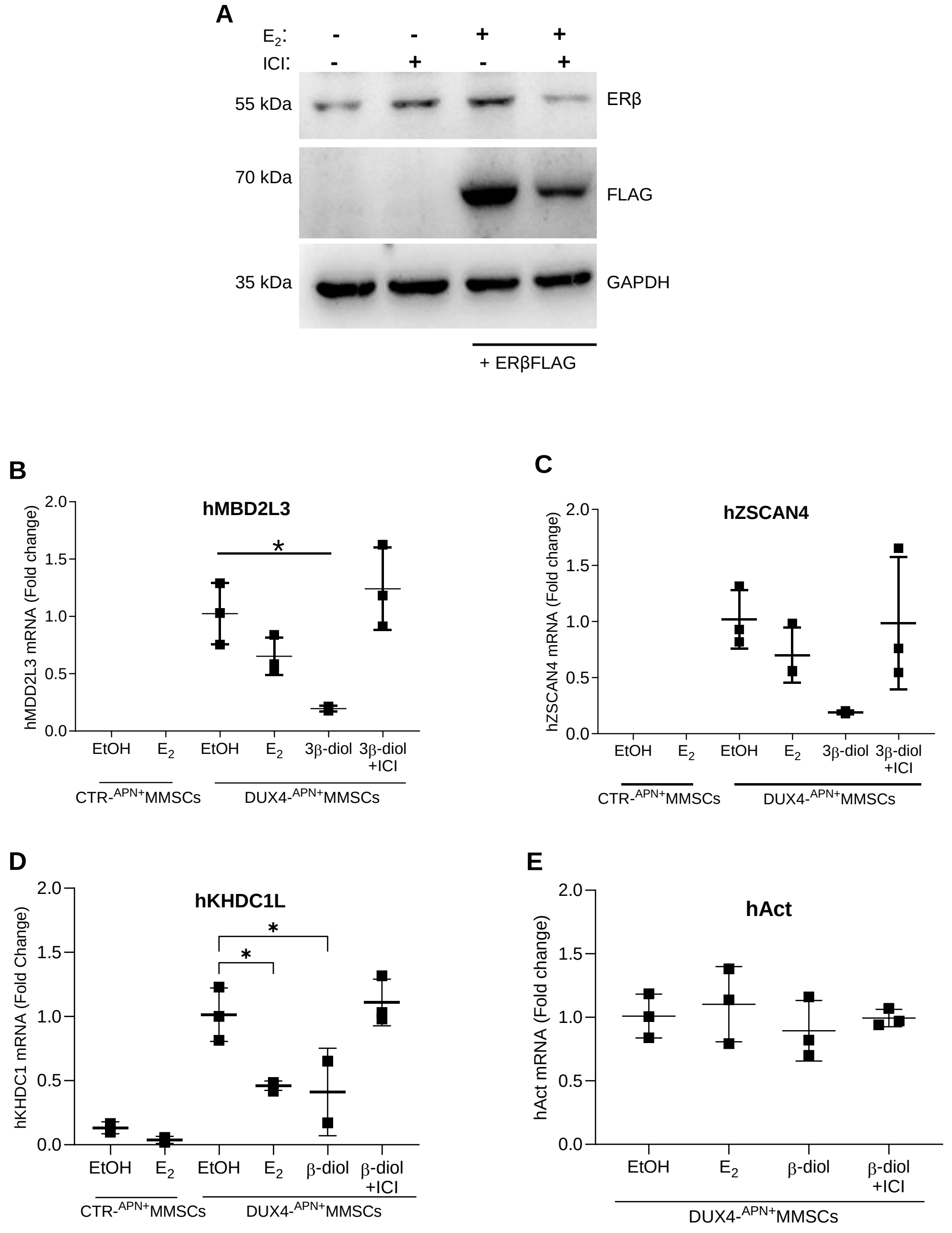

Fig. S3 Transcriptional activity of DUX4 in APN+MMSCs. A Degradative activity of ICI on endogenous or exogenous ERβ (ERβ-FLAG) in human immortalized myoblasts in the presence of E_2**.** Cells were treated as indicated and cell lysates collected after 48 hrs. **B-E** Human MBD2L3 (**B**)**,** ZSCAN (**C**) KHDC1L (**D**) and actin (**E**) mRNA levels by RT-qPCR in CTR-APN+MMSCs or DUX4-APN+MMSCs grafts, treated as indicated. Animals were sacrificed 8 days after transplantation. Samples were normalized to hActin (**B**-**C**) or hGAPDH (**D**-**E**) and relative to EtOH-treated DUX4-APN+MMSCs grafts, whose mean was arbitrarily set to 1. N=3 mice/group. Data is shown as mean ± SD. *$P < 0.05$, One-way ANOVA with Dunnet's multiple comparison test.

Fig. S4

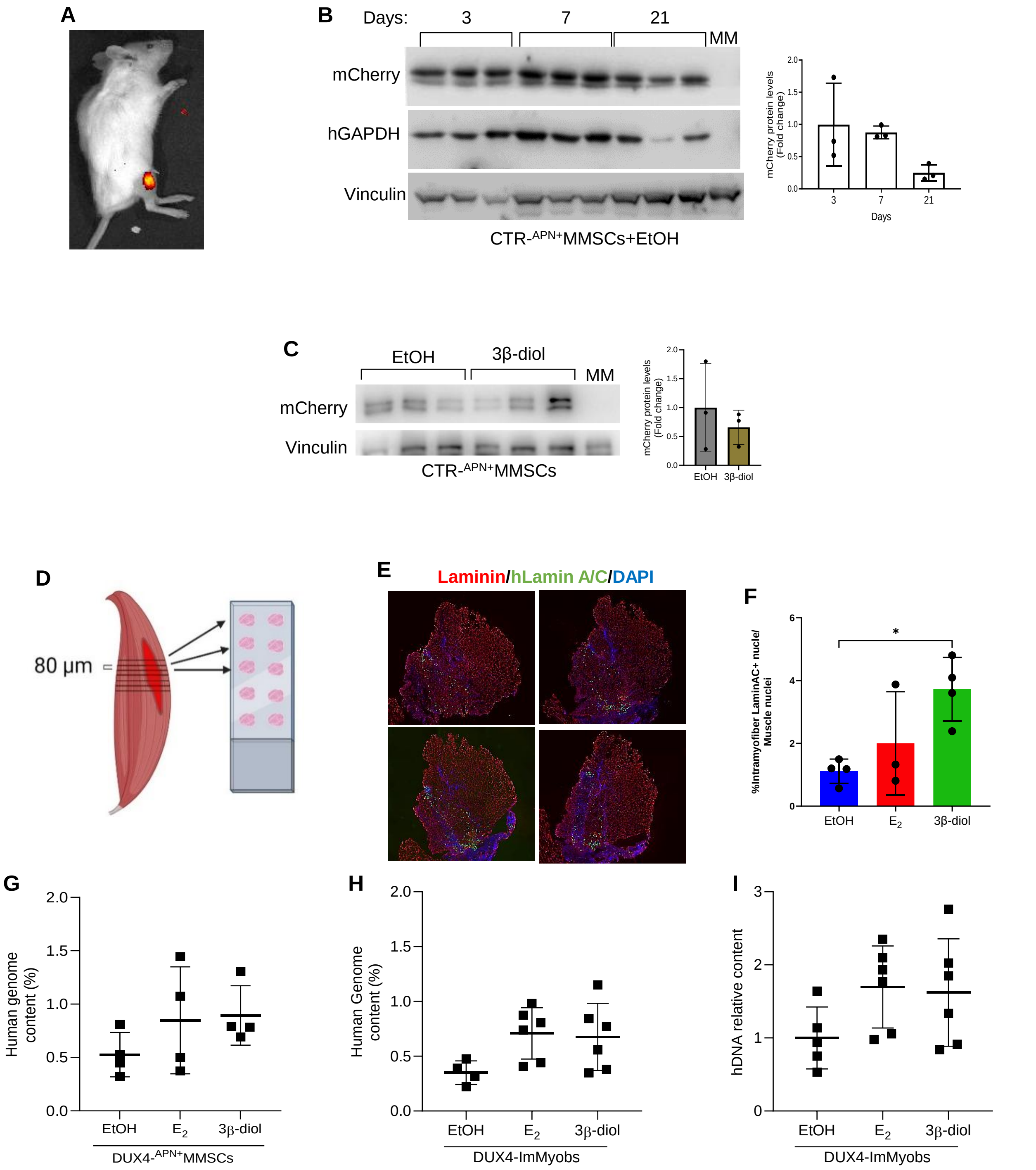

Fig. S4 Estrogen activity on survival of engrafted human cell. A Representative image of mCherry signal in the TA of NGS mice transplanted with mCherry-$^{APN+}$MMSCs. **B-C** WB of mCherry protein levels in TA muscles of male mice transplanted with CTR-$^{APN+}$MMSCs. Animals were sacrificed at the indicated time points (**B**) or four weeks (**C**) after transplantation. The graphs on the right report the relative quantification of protein levels. All samples were normalized to murine Vinculin. Naïve murine muscle (MM) was used as control. n=3 mice/group. **D** Scheme of collection of transversal sections for each TA muscle. Consecutive transversal sections - 10µm thickness and distant 80 µm each other - encompassing at least 300 µm length around the transplantation site were collected and used for IMF and histological analysis. **E** Representative images of sequential sections of TA muscle transplanted with CTR-$^{APN+}$MMSCs. **F** Percentage of human nuclei integrated in myofibers in sections of male DUX4-APN^{+}MMSCs grafts, treated as indicated. Each data point is the mean of four different sections/murine TA encompassing the transplantation site (according to the scheme in Fig. S4D). Animals were sacrificed four weeks following transplantation. n=3 mice/group. Data is shown as mean ± SD, $^{**}P < 0.01$, , One-way ANOVA with Dunnett's multiple comparisons test. **G-I** Human DNA quantification by qPCR in TRIzol DNA-fractions of DUX4-$^{APN+}$MMSCs (**G**) or DUX4-ImMyobs male grafts (**H,I**), treated as indicated and sacrificed after four weeks. Human genome content percentage was quantified using FOXP1 gene primer pairs (**G-H**). Human DNA relative content was quantified by human TITIN normalized to murine PTGER2 and relative to EtOH-treated DUX4-cells (**I**): **G**: n=4 mice/group; **H-I**: n_{EtOH}=5, n_{E2}, $n_{3b\text{-}diol}$=6

Fig. S5

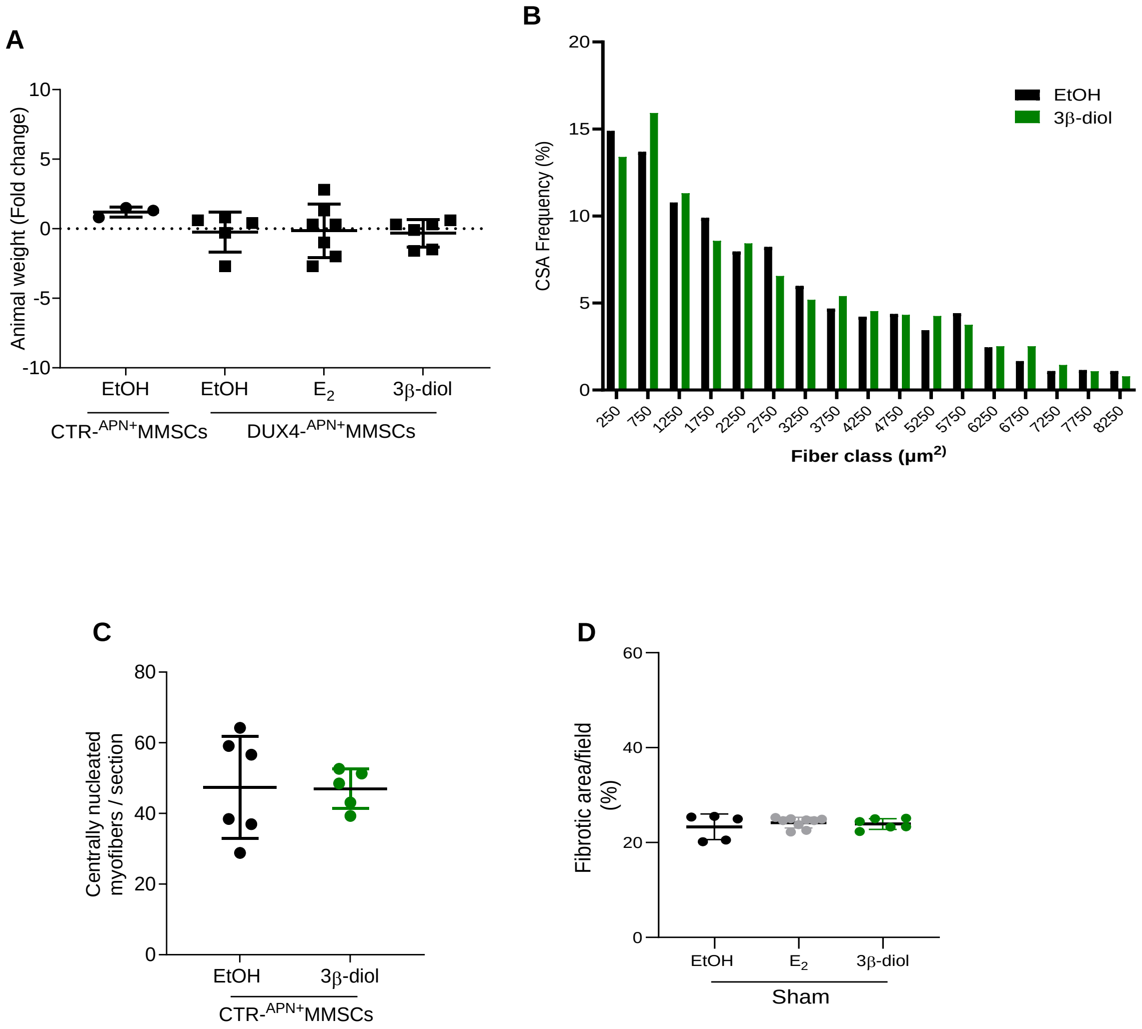

Fig. S5 Estrogen activity towards engrafted murine muscle. **A** Weight of mice treated as indicated. Animal weight was recorded at the beginning and the end of the treatment before the sacrifice. The weight is reported as fold change to the starting weight. $n_{CTR\text{-}APN+MMSCs}$ =3, $n_{DUX4\text{-}APN+MMSCs}$=5. **B** Quantification of CSA frequency in murine TA muscle of male mice engrafted with CTR-$^{APN+}$MMSCs and treated as indicated. Animals were sacrificed four weeks after transplantation. n=6 sections/mouse, 2 mice/group . Data is shown as mean ± SD. **C** Quantification of CNF (centrally nucleated myofibers, containing at least 1 central nucleus) in males CTR-$^{APN+}$MMSCs grafts. Animals were sacrificed four weeks after transplantation. n=3 sections/mouse 2 mice/group. **D** Quantification of muscle fibrosis in TA muscle of male mice transplanted with Matrigel (Sham) and treated as indicated. Animals were sacrificed four weeks after transplantation. Data is expressed as % of red picrosirius^{+} pixels to the total pixel number (red+yellow). Data is from 1 experimental group. n=3 sections/mouse, n_{EtOH}=5 mice, n_{E2}=9 mice $n_{3\beta\text{-}diol}$=6 mice Data is shown as mean ± SD.

Fig. S6

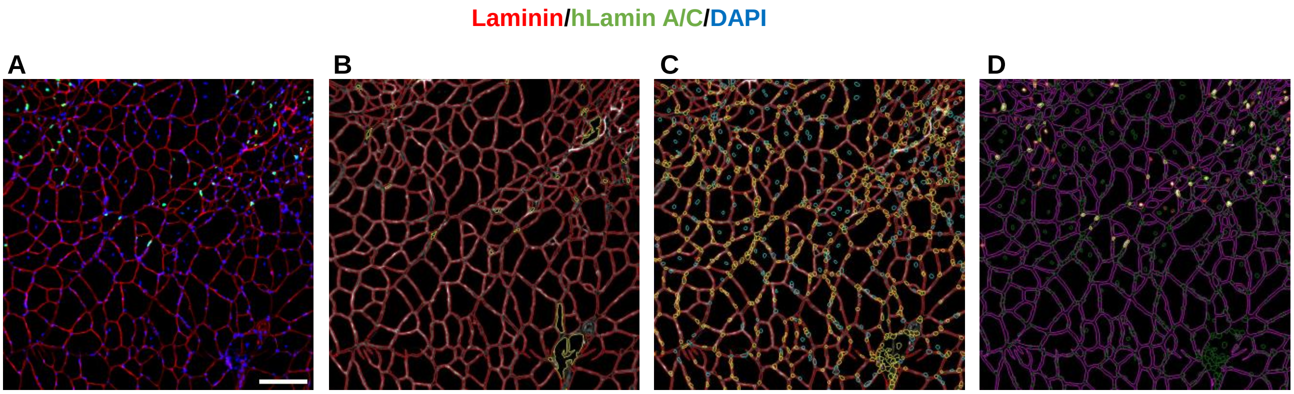

Fig. S6 "MyoEngraftmentProfiler" pipeline developed with CellProfiler software. **A** Representative cropped immunofluorescence image of the TA muscle cross-section, displaying Laminin (red), human Lamin A/C (green) and DAPI (blue). Scale bar is 100 µm. **B** Segmentation of muscle fibers (red) outlined on grayscale Laminin image. Discarded muscle fibers are outlined in yellow. **C** Segmentation of muscle nuclei (cyan) and non-muscle nuclei (i.e. stromal/interstitial nuclei; yellow). Muscle fibers are outlined in red. **D** Segmentation of Lamin A/C-positive nuclei (i.e. human nuclei; yellow) and Lamin A/C-negative nuclei (i.e. murine nuclei; green). Muscle fibers are outlined in magenta.

Table S1. Features of FSHD patients and CTR individuals

Patient	Sex	Biopsy side[a]	Fragment length (D4Z4 units)		CSS	Age at biopsy
**FSHD1#1**	Female	Quadr dx	5		4	52
**FSHD1#2**	Female	Vast.Lat.dx	8		0	51
**FSHD1#5**	Female	Delt.	6		4	55
**CTR#1**	Female	Quadr				87
**CTR#2**	Female	Delt				68
**CTR#5**	Male	For				20
**CTR#11**	Female	PSM				73
**CTR#12**	Female	PSM				74
**CTR#13**	Male	PSM				62
**CTR#15**	Male	Glu max				77

[a] Quadr.= quadriceps; Vast. Lat.=vastus lateralis; Delt.= deltoides; For=Forearm; PSM= Paraspinal muscle; Glu. Max= Gluteus maximus

Table S2. **Primers and probes used for qPCR**

Target gene	Primer sequence 5’-3’/assay	Probe/SYBR Green
TRIM43	Fw ACCCATCACTGGACTGGTGT<br>Rev CACATCCTCAAAGAGCCTGA	SYBR Green
ZSCAN4	Fw TGGAAATCAAGTGGCAAAAAC<br>Rev CTGCATGTGGACGTGGAC	SYBR Green
LEUTX	Fw GACCATGCACCCAAGTTTGG<br>Rev GGTTTGCTGGCCCTAGTGAT	SYBR Green
MBD2L3	Fw AGCTATGGGAGAACCTGCGT<br>Rev TCATGGGGAGAGCAGACCTC	SYBR Green
KHDC1L	Fw TGAATCAGGTGGGAGCACAG<br>Rev CAATGCAGCGAAGGTACGTG	SYBR Green
mWfdc3	Mm01243777_m1	Probe (Thermo)
mGapdh	Mm99999915_g1	Probe (Thermo)
DUX4	Fw CCAAGGTACCAGCAGACC<br>Rev TCCAGGAGATGTAACTCTAATCCA	SYBR Green
ESR2	Fw CACTTCATGTTGAGCAGATGTTC<br>Rev TCTCCTCCCAGCAGCAAT	Probe (IDT)
ESR1	Fw GAACCGAGATGATGTAGCCA<br>Rev GTTTGCTCCTAACTTGCTCTTG	Probe (IDT)
hTITIN	Fw ACCACATGCATTTTATCAGAGC<br>Rev GCCTGTTGAGAATGTTCAAGG	SYBR Green
mPtger2	Fw CCTGCTGCTTATCGTGGCTG<br>Rev CTCCGACGGTGCATGCGA	SYBR Green
hGAPDH	Fw ATGGGGAAGGTGAAGGTC<br>Rev TCCCGTTCTCAGCCTTGAC	SYBR Green
hACTIN	Fw CGCCGCCAGCTCACC<br>Rev CACGATGGAGGGGAA	SYBR Green
hRPLPO	Fw TGTCTGCTCCCACAATGAAAC<br>Rev TCGTCTTTAAACCCGCGTG	Probe (IDT)
mGapdh	Fw CAATGAATACGGCTACAGCAAC<br>Rev AGGGAGATGCTCAGTGTTGG	SYBR Green
mActin	Fw CCAGTTGGTAACAATGCCATGT<br>Rev GGCTGTATTCCCCTCCATCG	SYBR Green
FOX P1 (Human+Mouse)	Fw CGTATGACCGGCTTAACATCTCTA<br>Rev CAGTCACCTAAAACATGCAGCA	SYBR Green
FOX P1 (Human specific)	Fw CGTATGACCGGCTTAACATCTCTA<br>Rev CACCTAAAACATGCAGCAAAAGA	SYBR Green
Fox P1 (Mouse specific)	Fw CGTATGACCGGCTTAACATCTCTA<br>Rev CCTAAAACATGCAGCAGAAGC3’	SYBR Green

Table S3. Primary and secondary antibodies used for western blot, immunofluorescence, and flow cytometry

**Target protein (clone)**	**Catalogue number**	**Company**
ERβ (14C8)	GTX70174	Genetex
hERα (F-10)	sc-8002	Santa Cruz
ERα (D-12)	sc-8005	Santa Cruz
DUX4 (E5-5)	ab124699	Abcam
mCherry (16D7)	M11217	Invitrogen
Desmin (DE-R-11)	sc-58745	Santa Cruz
PDGFRβ (28E1)	3169	Cell signaling
Lamin A/C	PA1-41104	Invitrogen
Laminin	L9393	Sigma
hNG2 (LHM-2)	FAB2585A	Biotechne
hCD140a (αR1)	564594	BD Biosciences
Vinculin (V284)	05-386	Sigma
GAPDH (GA1R)	MA5-15738	Invitrogen
hGAPDH (0411)	sc-47724	Santa Cruz
**Secondary antibody**		
Alexa Fluor™ Plus 488	A32723	Invitrogen
Alexa Fluor™ Plus 594	A32740	Invitrogen
