# Supplementary material for: Estrogen rescues muscle regeneration impaired by DUX4 in a humanized xenograft mouse model
Source: Cell Death Dis. 2025 Jul 9;16(1):508. doi: 10.1038/s41419-025-07827-2 (PMC12241518; doi:10.1038/s41419-025-07827-2)
Supplement: Supplementary file 2 — Original Data [file 41419_2025_7827_MOESM2_ESM.pdf]

Full unedited gels for Fig. 4c (left panel)

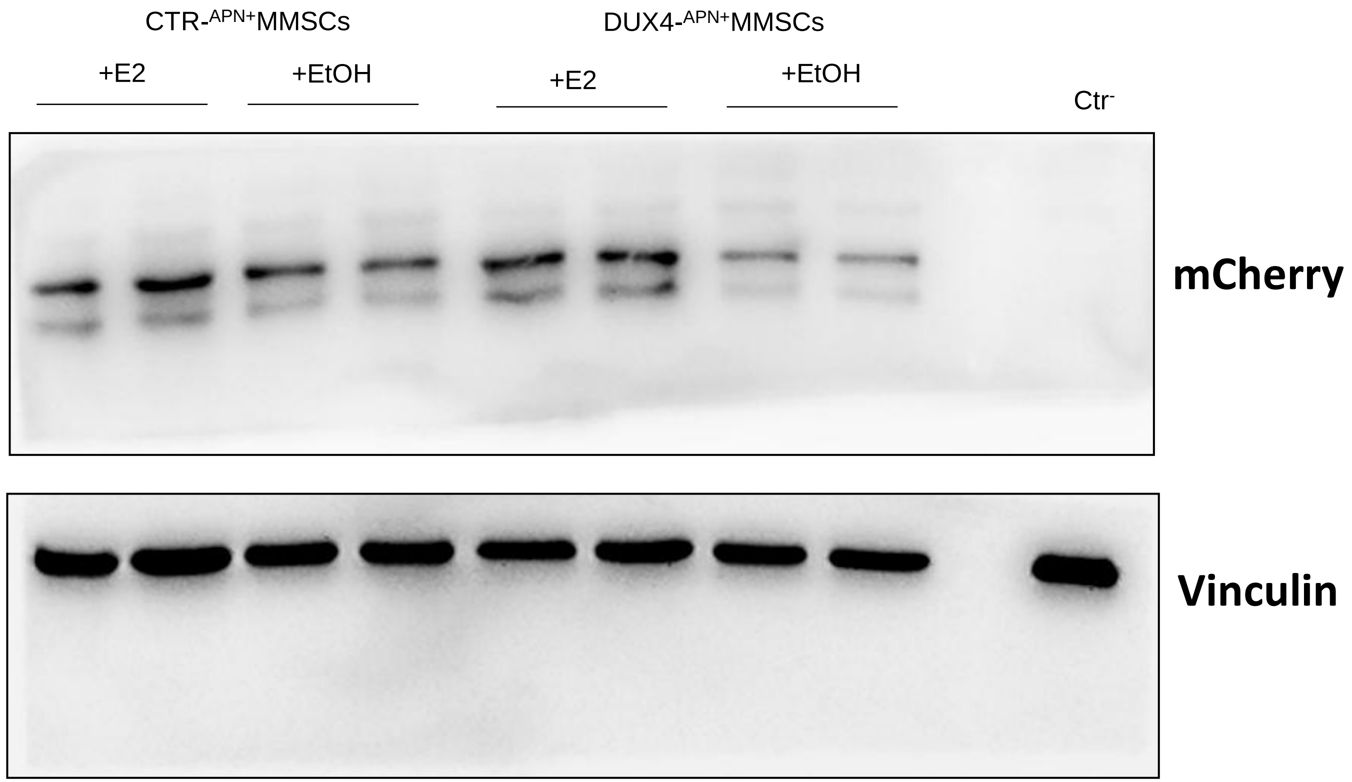

Full unedited gels for Fig. 4c (right panel)

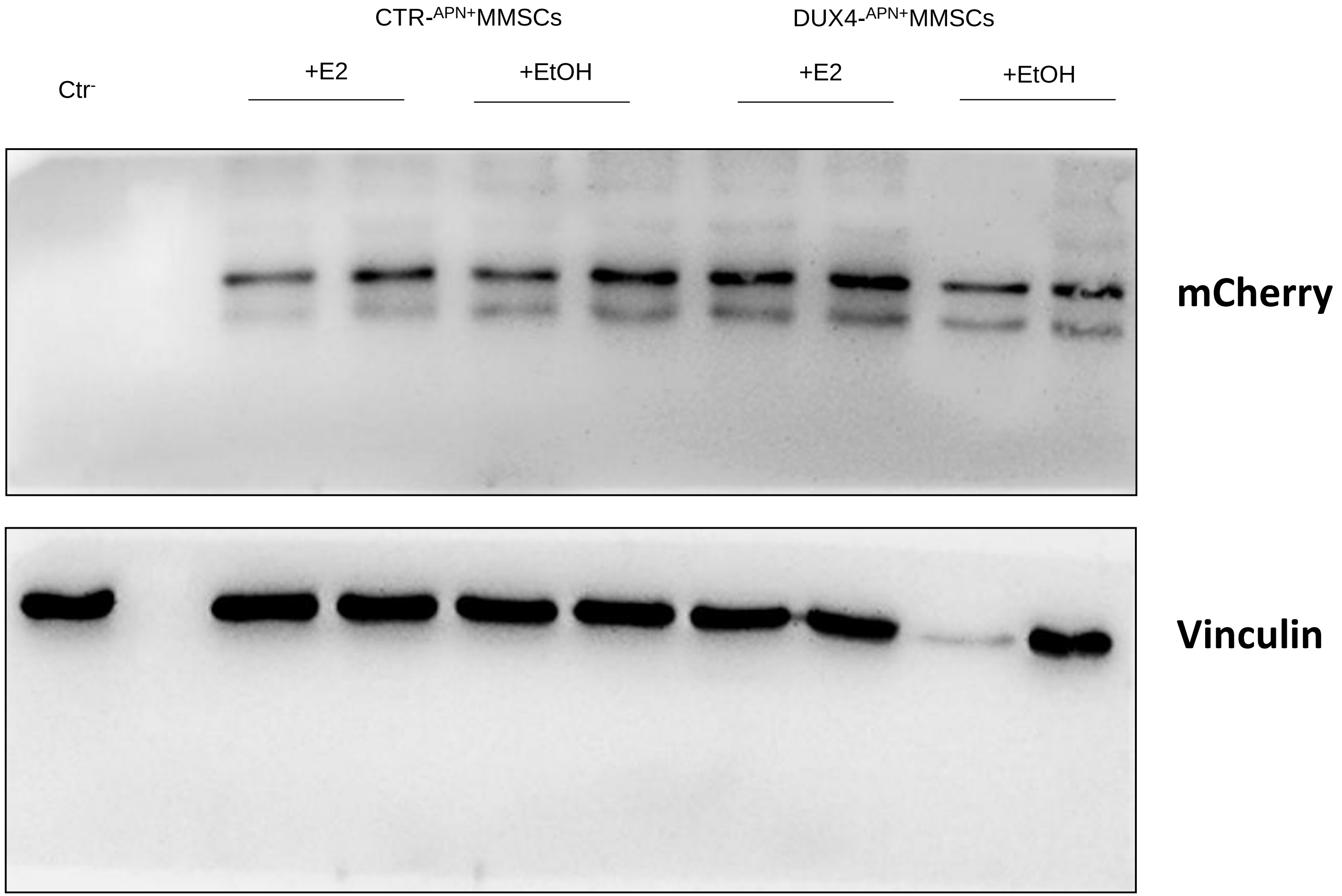

Full unedited gels for Fig. 4d

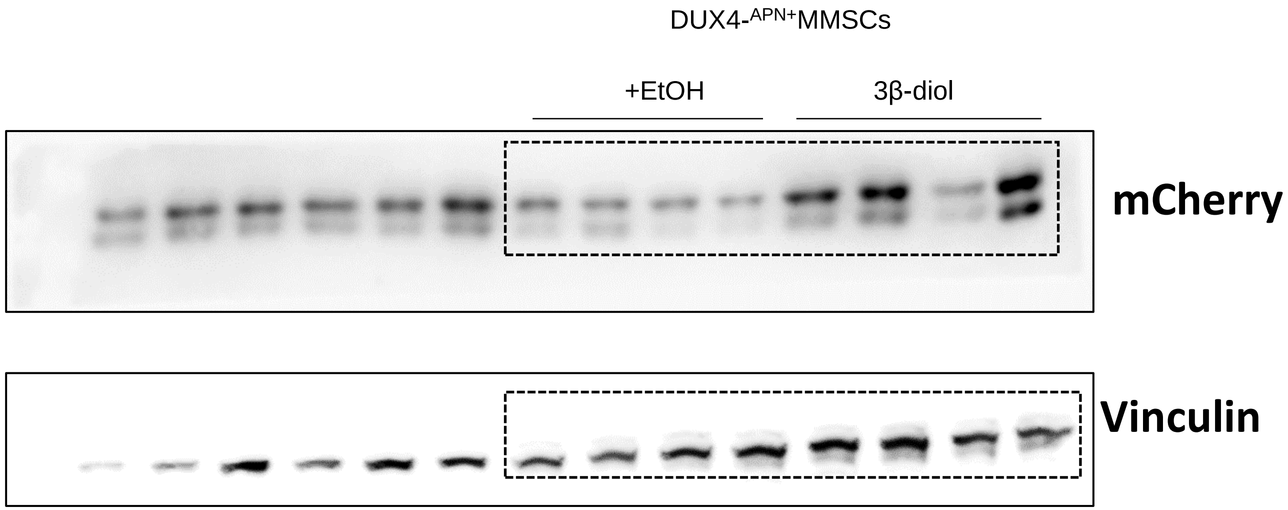

Full unedited gels for Fig. S1h

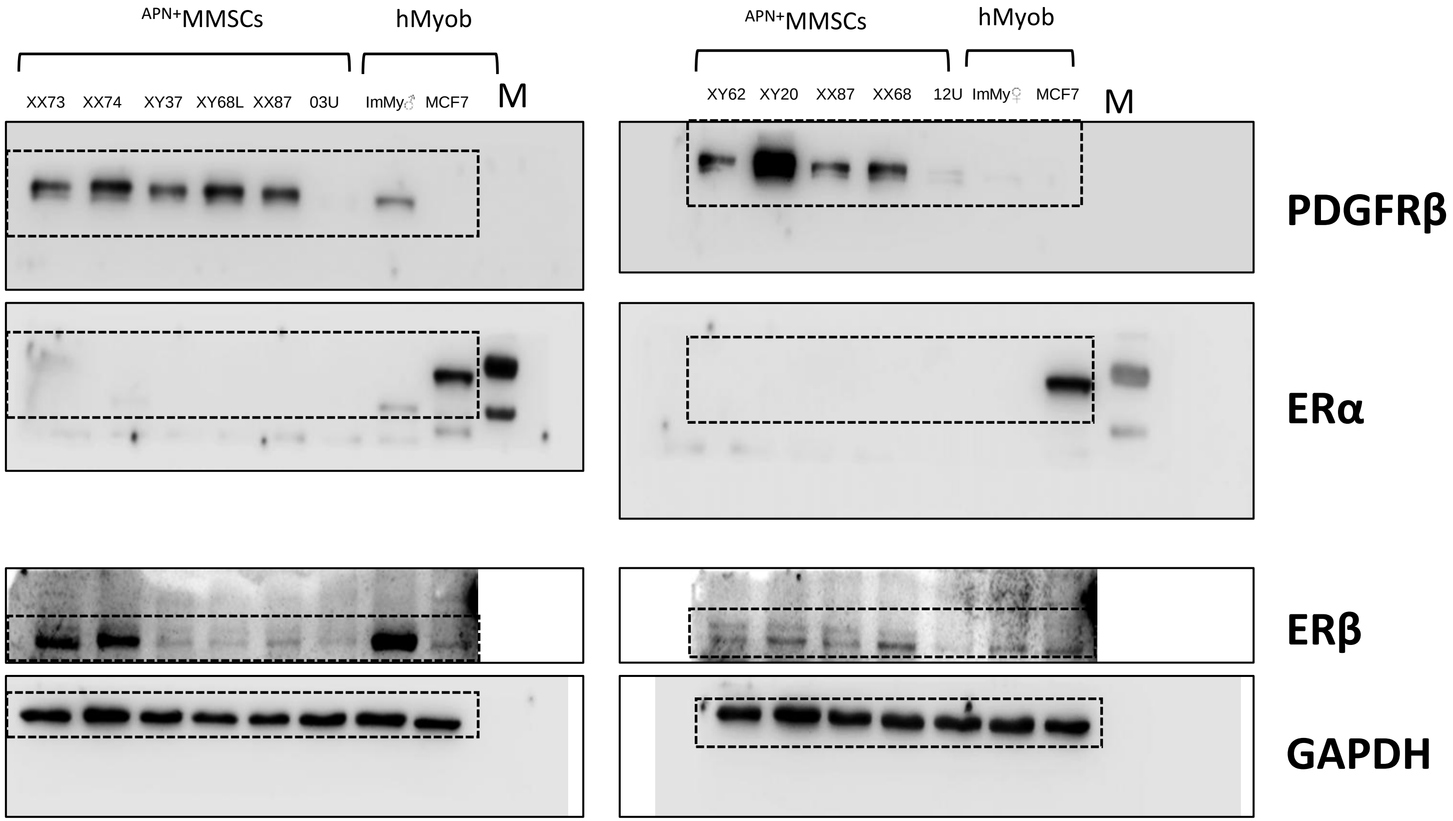

Full unedited gels for Fig. S3a

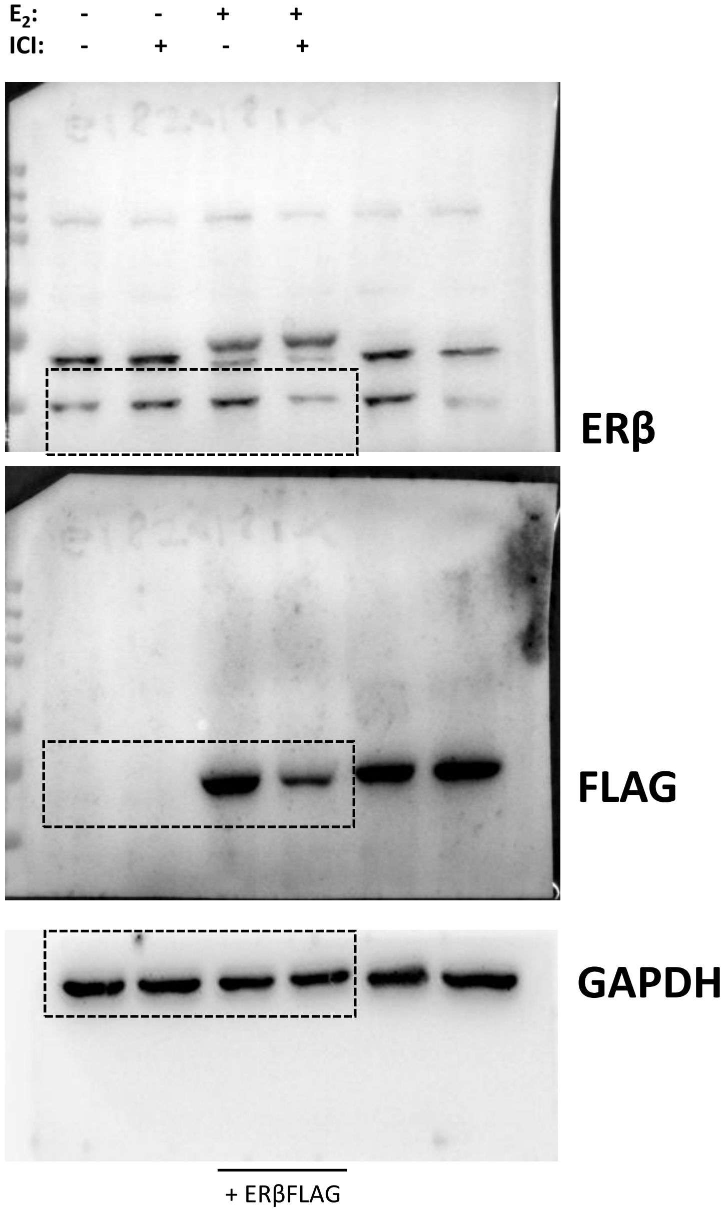

Full unedited gels for Fig. S4b

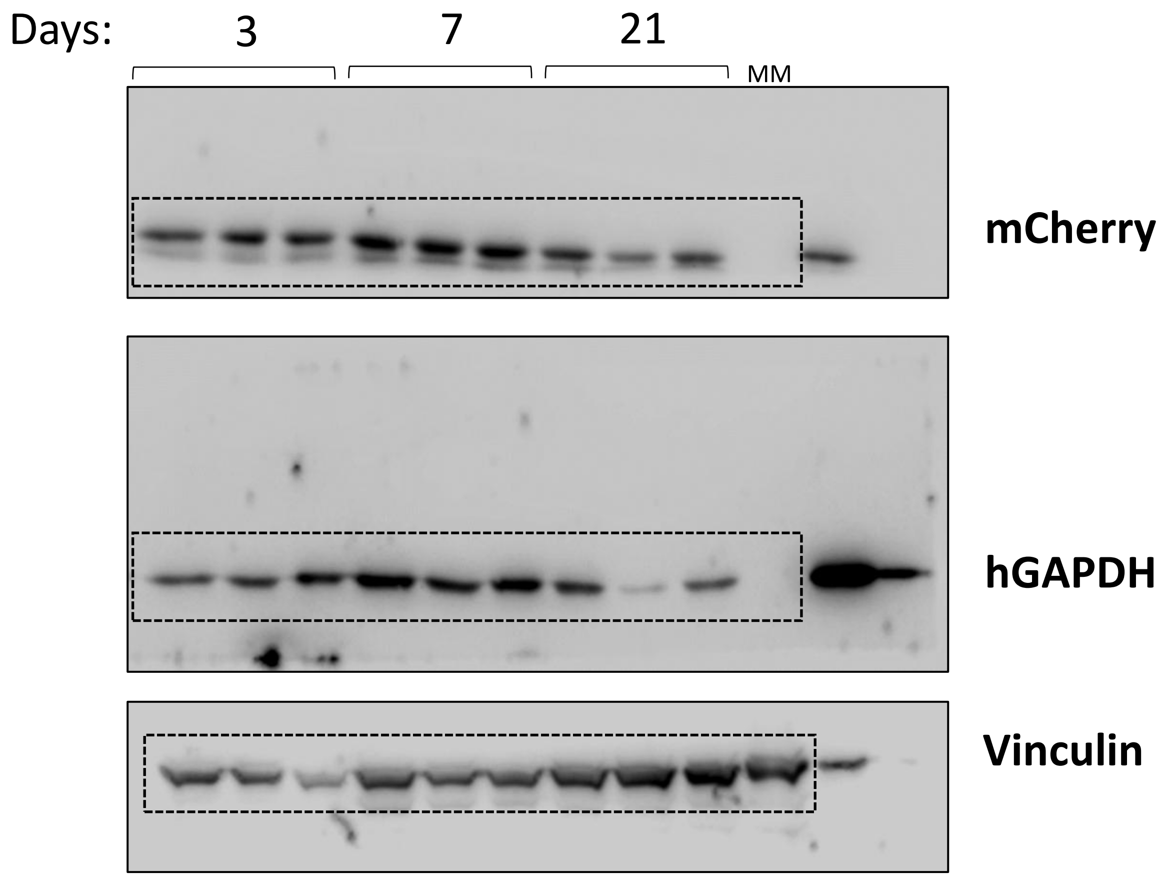

CTR-[APN+]MMSCs + EtOH

Full unedited gels for Fig. S4c

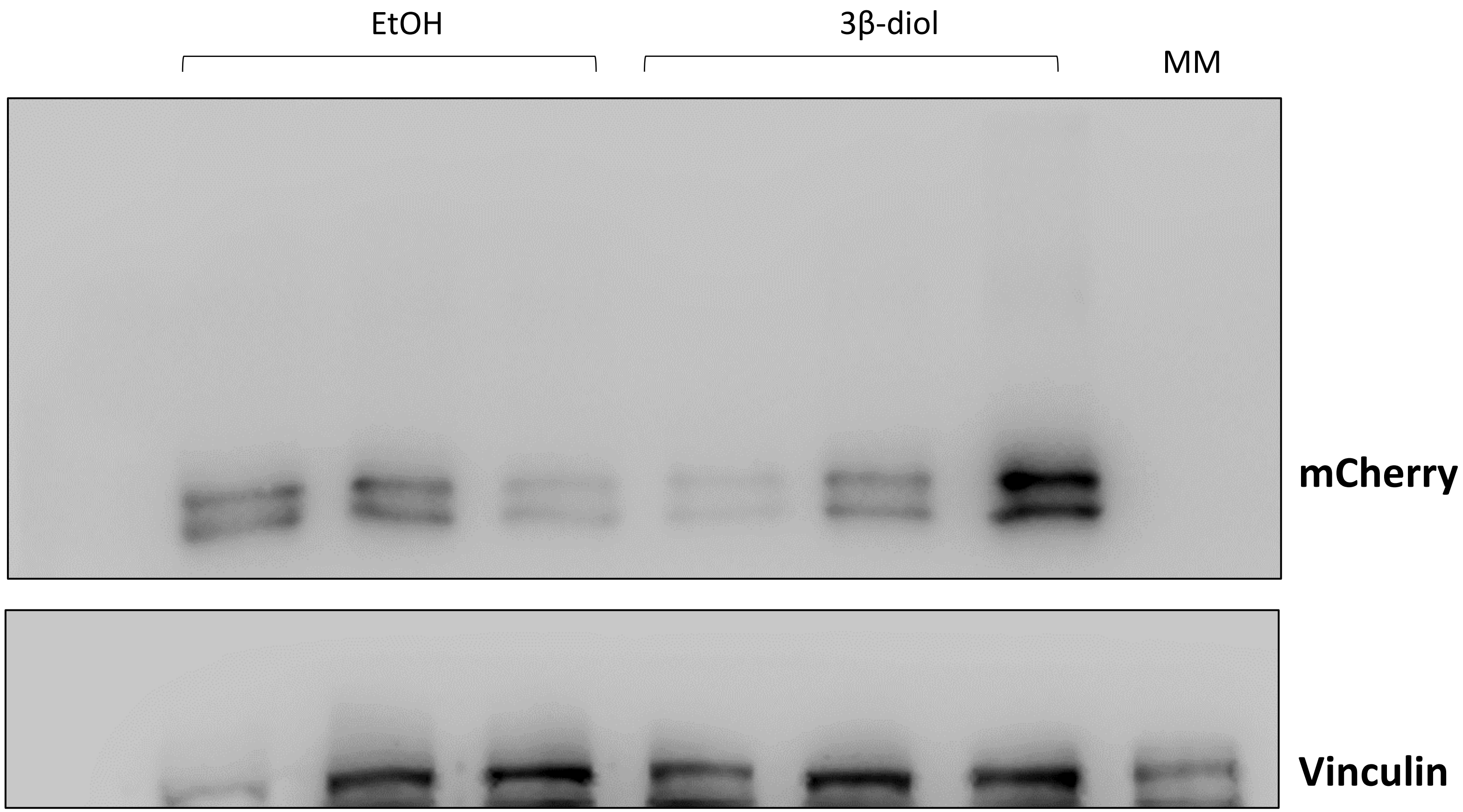

CTR-$^{APN+}$MMSCs+EtOH
